# Engineering of human mesenchymal stem cells resistant to multiple natural killer subtypes

**DOI:** 10.7150/ijbs.64640

**Published:** 2022-01-01

**Authors:** Dejin Zheng, Xiaoyan Wang, Zhenwu Zhang, Enqin Li, Cheungkwan Yeung, Roma Borkar, Guihui Qin, Yaojiong Wu, Ren-He Xu

**Affiliations:** 1 Center of Reproduction, Development & Aging, and Institute of Translational Medicine, Faculty of Health Sciences, University of Macau, Taipa, Macau, China.; 2 Ministry of Education Frontiers Science Center for Precision Oncology, University of Macau, Taipa, Macau, China.; 3 School of Life Science and Technology, ShanghaiTech University, Shanghai, China.; 4 The Shenzhen Key Laboratory of Health Sciences and Technology, International Graduate School at Shenzhen, Tsinghua University, Shenzhen, China.

**Keywords:** Human embryonic stem cells, mesenchymal stem cells, natural killer cells, innate immunity, immune rejection

## Abstract

Mesenchymal stem cells (MSCs) as a therapeutic promise are often quickly cleared by innate immune cells of the host including natural killer (NK) cells. Efforts have been made to generate immune-escaping human embryonic stem cells (hESCs) where T cell immunity is evaded by defecting β-2-microglobulin (B2M), a common unit for human leukocyte antigen (HLA) class I, and NK cells are inhibited via ectopic expression of *HLA-E* or *-G*. However, NK subtypes vary among recipients and even at different pathologic statuses. It is necessary to dissect and optimize the efficacy of the immune-escaping cells against NK subtypes. Here, we first generated *B2M* knockout hESCs and differentiated them to MSCs (EMSCs) and found that NK resistance occurred with *B2M^-/-^* EMSCs expressing *HLA-E* and *-G* only when they were transduced via an inducible lentiviral system in a dose-dependent manner but not when they were inserted into a safe harbor. HLA-E and -G expressed at high levels together in transduced EMSCs inhibited three major NK subtypes, including *NKG2A^+^*/*LILRB1^+^*, *NKG2A^+^*/*LILRB1^-^*, and *NKG2A^-^*/*LILRB1^+^*, which was further potentiated by IFN-γ priming. Thus, this study engineers MSCs with resistance to multiple NK subtypes and underscores that dosage matters when a transgene is used to confer a novel effect to host cells, especially for therapeutic cells to evade immune rejection.

## Introduction

Mesenchymal stem cells (MSCs) exert promising therapeutic effects on many disease models mainly through secreted factors and exosomes for immunomodulation and regeneration, and in a rarer chance via direct differentiation. However, their outcomes in clinical trials often fluctuate 1, which might partially result from clearance of allogeneic MSCs infused either systemically or locally, due to both innate immunity immediately and adaptive immunity at a later time 2-4. T cell-mediated adaptive immunity plays an essential role in the clearance of allogeneic MSCs 2, which is triggered by allogeneic peptides presented on the cell surface by human leukocyte antigens (HLAs) 5. Many strategies have been developed to reduce the immunogenicity of MSCs. Classical HLA class I (HLA-Ia) is the primary target of manipulation due to its important role in MSC immunogenicity. β-2-microglobulin (*B2M*) knockout MSCs were generated from umbilical cord MSCs and MSCs differentiated from induced pluripotent stem cells (iPSCs). *B2M^-/-^* MSCs became more resistant to MSC-primed CD8^+^ T cell-mediated lysis even after IFN-γ priming 6-8. In addition, *B2M^-/-^* MSCs also had better therapeutic effects for treating myocardial infarction and ischemic hindlimb compared to wild-type (WT) MSCs 7, 8.

However, since HLA-Ia molecules are the classical ligands for the killer-cell immunoglobulin-like receptors (KIRs) on natural killer (NK) cells, a major type of innate immune cells 9, removal of HLA-Ia from any cells including MSCs sensitizes the cells to NK cytotoxicity 6. To solve this problem, MSCs were derived from HLA-Ia pseudo-homozygous iPSCs with monoallelic deletion of *HLA-A*, *B*, and *C* genes to avoid NK cytotoxicity 10. However, this strategy still requires HLA matching to evade adaptive immunity. Unlike HLA-Ia, non-classical HLA class I molecules (HLA-Ib) such as HLA-E or HLA-G barely activate adaptive immunity, while still protecting cells from NK cell-mediated lysis through interacting with NK inhibitory receptors including natural killer group 2 member A (NKG2A)/CD94 and leukocyte immunoglobulin-like receptor subfamily B member 1 (LILRB1) 11, 12.

Strategies based on HLA-Ib have been developed to evade NK immunity in HLA-null human pluripotent stem cells (hPSCs), including embryonic stem cells (ESCs) and iPSCs, which have led to the generation of so-called universal hPSCs 13. For example, Gornalusse, *et al.*, inserted a *B2M-HLA-E* fusion gene into the *B2M* loci of hESCs using an adeno-associated virus vector. The resultant hESCs and their derived hematopoietic cells can escape the allogeneic responses and NK cell-mediated killing *in vitro* and *in vivo* 14. Moreover, HLA-G expression in HLA-Ia-depleted hESCs also protect against NK cell-mediated lysis 15. Co-expression of B2M-fused HLA-G1 and -G5 proteins in *B2M* deficient hESCs has potent inhibitory effects on both T and NK cells 16. Nevertheless, it awaits to be tested whether such strategies remain effective in hPSC-derived MSCs. Moreover, NK cells are heterozygous with a donor-dependent profile of their receptor expression 17, and the reported approaches only inhibit limited NK subtypes. For example, cells bearing HLA-E or -G alone only escape *NKG2A^+^* or* LILRB1^+^* NK subtypes, respectively 14, 16. Therefore, it is necessary to develop MSCs with combined resistance to multiple NK subtypes.

In this study, we first knocked out *B2M*, a common unit for all class I HLAs, in hESCs and then inserted a single copy of engineered *HLA-E* and *-G* together in the safe harbor locus *AAVS1* in the genome of the *B2M^-/-^* hESCs, which were then differentiated to MSCs (EMSCs). Unexpectedly, the genetically manipulated EMSCs were still sensitive to human NK cytotoxicity. However, when *HLA-E* or *-G* were expressed via a doxycycline (DOX)-inducible lentiviral system, NK resistance was achieved with high doses of DOX, suggesting that NK cytotoxicity can be escaped only when ectopically expressed immunosuppressive molecules reach a threshold. Based on these, we generated EMSCs with the capability to resist multiple NK subtypes including *NKG2A^+^*/*LILRB1^+^*, *NKG2A^+^*/*LILRB1^-^*, and *NKG2A^-^*/*LILRB1^+^* NK cells.

## Results

### *B2M^-/-^* EMSCs are sensitized to NK cytotoxicity

**Figure 1:**
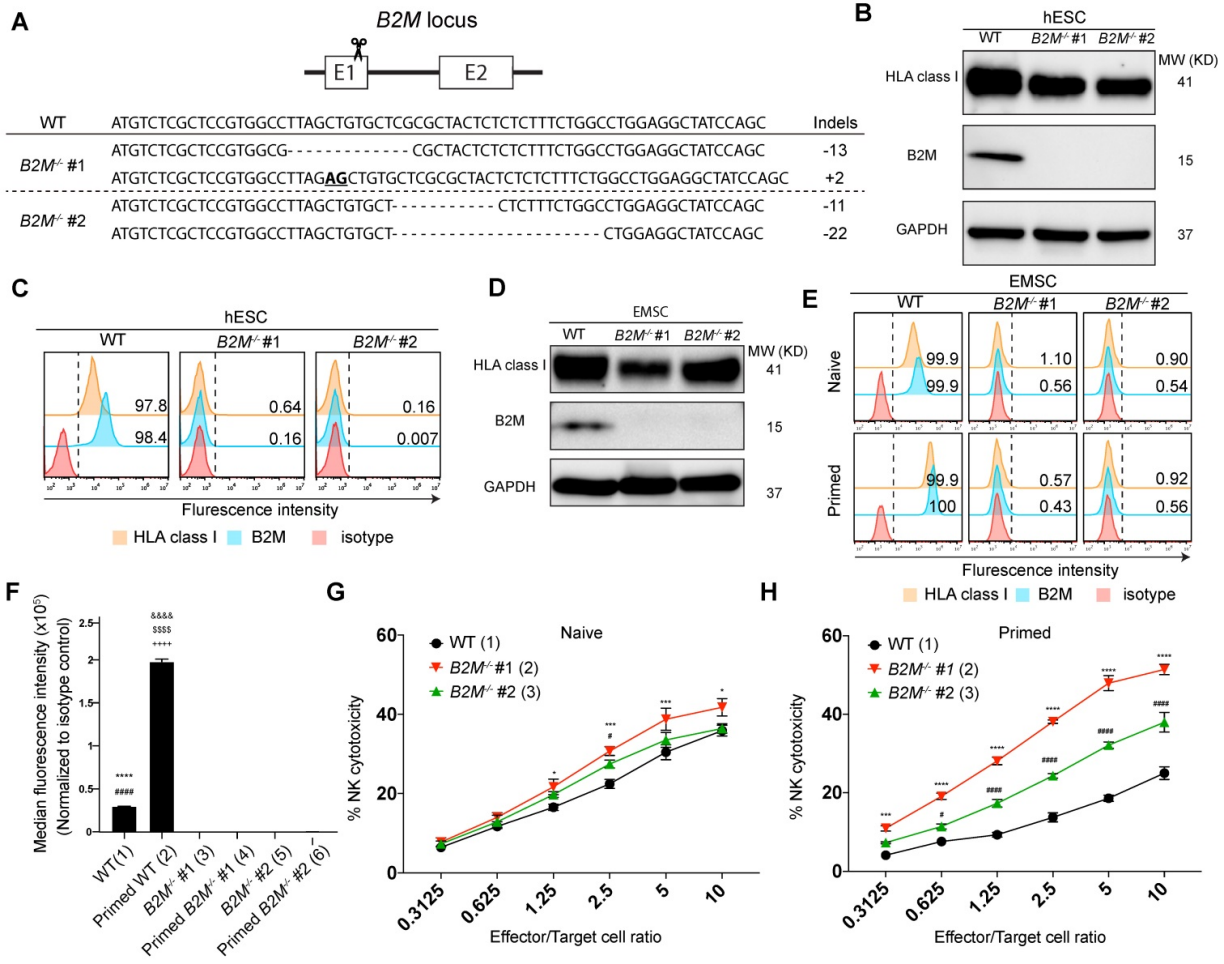
***B2M^-/-^* EMSCs are sensitized to NK cytotoxicity. (A)** Schematic graph and genotyping results for *B2M^-/-^* hESC clones #1 and 2 compared to WT control. **(B)** Western blotting for HLA class I and B2M expression in WT and *B2M^-/-^* hESCs. GAPDH was tested as an internal control. **(C)** Flow cytometry analysis for HLA class I and B2M expression on the surface of WT and *B2M^-/-^* hESCs. **(D)** Western blotting for HLA class I and B2M expression in WT and *B2M^-/-^* EMSCs. GAPDH was tested as an internal control. **(E)** Flow cytometry analysis for B2M and HLA class I expression on the surface of WT and *B2M^-/-^* EMSCs treated with (IFN-γ-primed) or without (naïve) 10 ng/ml IFN-γ. **(F)** Summary of the medium fluorescence intensities for the above results. N = 3, ^****^P < 0.0001 for (1) versus (3), ^####^*P* < 0.0001 for (1) versus (5), ^&&&&^*P* < 0.0001 for (2) versus (4), ^$$$$^*P* < 0.0001 for (2) versus (6), ^++++^*P* < 0.0001 for (2) versus (1) EMSCs per ordinary one-way ANOVA followed by Tukey's multiple comparison test. **(G)** NK-92MI cell-mediated lysis against WT and *B2M^-/-^* EMSCs (naïve). N = 3, ^*^P < 0.05, ^***^P < 0.001 for (2) versus (1), ^#^*P* < 0.05 for (3) versus (1) per two-way ANOVA followed by Dunnett's multiple comparison test. **(H)** NK-92MI cell-mediated lysis against WT and *B2M^-/-^* EMSCs (IFN-γ-primed). N = 3, ^***^P < 0.001, ^****^P < 0.0001 for (2) versus (1); ^#^*P* < 0.05, ^####^*P* < 0.0001 for (3) versus (1) per two-way ANOVA followed by Dunnett's multiple comparison test.

To develop EMSCs that can evade T cell immunity, we first targeted *B2M* gene 18 in H1 hESC line using the clustered regularly interspaced short palindromic repeats (CRISPR)/CRISPR-associated protein 9 (Cas9) technology with a pair of single guide (sg) RNAs specifically for *B2M* and obtained two *B2M^-/-^* cell clones with frame-shift mutations in exon 1, *i.e.*, -13/+2 and -11/-22, respectively, based on genotyping (Fig. 1A). As expected, western blotting detected B2M expression in the WT control but not the two *B2M^-/-^* clones. On the other hand, the total HLA class I expression was detected in the WT control and, to a lesser level, in the *B2M^-/-^* clones (Fig. 1B). Flow cytometry confirmed that both B2M and HLA class I were present on the surface of the WT control but not of the *B2M^-/-^* hESCs (Fig. 1C). The genome-editing didn't affect the pluripotency of the hESCs based on assays for expression of pluripotency marker genes (Fig. S1A) and teratoma formation (Fig. S1B). Normal karyotypes were also preserved in the genetically manipulated hESC clones (Fig. S1C).

Further, we differentiated *B2M^-/-^* hESCs to MSCs using an established protocol 19, 20 (Fig. S2A). The resultant EMSCs, like the WT control, met the minimum criteria for MSCs based on their positivity of CD90, CD44, CD105, and CD73 and negativity of CD34, CD45, CD11b, CD19, and HLA-DR (Fig. S2B) and their capability of trilineage differentiation to adipocytes, osteoblasts, and chondrocytes *in vitro* (Fig. S2C). Consistently, B2M expression was detected in the WT but not *B2M^-/-^* EMSCs using western blotting, while the total HLA class I expression was detected in both WT and *B2M^-/-^* EMSCs (Fig. 1D). Moreover, both B2M and HLA class I were present on the surface of the WT but not the *B2M^-/-^* EMSCs no matter whether the cells were primed with or without 10 ng/ml interferon-gamma (IFN-γ) (Figs. 1E and F).

To test whether the absence of HLA class I on the cell surface would sensitize the EMSCs to NK cells, we set up NK cytotoxicity assay *in vitro* using NK-92MI cells as effectors 21. *B2M^-/-^* EMSCs, as target cells, were more susceptive than WT EMSCs to the NK cell-mediated killing (Fig. 1G). It has been known that IFN-γ priming increases the resistance of MSCs to NK cells through elevated expression of HLA class I molecules 22. However, IFN-γ treatment increased the surface expression of HLA class I only in the WT EMSCs but not in the *B2M^-/-^* EMSCs (Figs. 1E and F). Consistently, following IFN-γ treatment, the WT but not the *B2M^-/-^* EMSCs had reduced sensitivity to the NK cytotoxicity compared to the unprimed controls (Figs. 1G and H). These results suggest that the absence of HLA class I on the cell surface sensitizes EMSCs to NK cytotoxicity.

### *HLA-E* and *-G* fused with *B2M* are inserted in a safe harbor locus in *B2M^-/-^* hESCs

**Figure 2:**
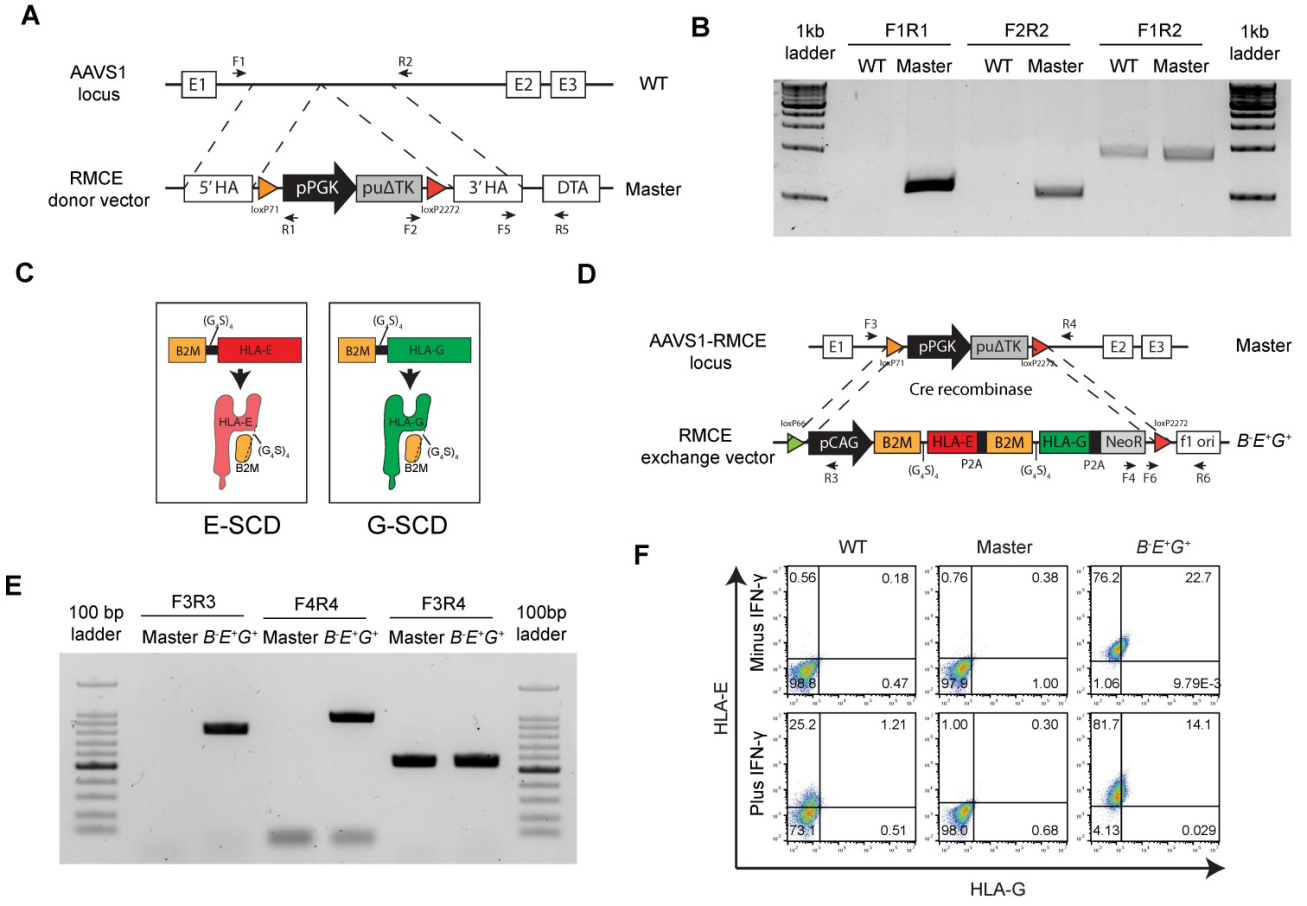
***B2M*-fused *HLA-E* and *-G* are inserted in a safe harbor locus in *B2M^-/-^* hESCs. (A)** Schematic graph for generation of RMCE master line in *B2M^-/-^* hESCs. A RMCE cassette containing floxed *PGK* promoter driven *puΔTK* is inserted into the *AAVS1* locus using TALENs. **(B)** PCR confirmation of stable integration of the RMCE cassette in the *AAVS1* site of master hESCs. PCR products were amplified from genomic DNA in WT and master hESCs using primes as indicated in A. **(C)** Schematic graph for a single-chain dimer (SCD) of HLA-E (E-SCD) or -G (G-SCD), which fused B2M with HLA-E or -G using a linker (G_4_S)_4_. **(D)** Schematic graph for knock-in of a cassette including *CAG* promoter-driven *E-SCD*, *G-SCD*, and *NeoR* from the RMCE donor vector into the *AAVS1* locus of *B2M^-/-^* hESCs via Cre-mediated RMCE. **(E)** PCR confirmation of the integration of *E-SCD*, *G-SCD*, and *NeoR* in the *AAVS1* site of *B^-^E^+^G^+^* hESCs. PCR products were amplified from the genomic DNA of master and *B^-^E^+^G^+^* hESCs using primes as indicated in D. **(F)** Flow cytometry analysis for HLA-E and -G expression on the surface of WT, master, and *B^-^E^+^G^+^* hESCs (with or without IFN-γ treatment).

To reduce the sensitivity of *B2M^-/-^* EMSCs to NK cytotoxicity, we expressed two *HLA-Ib* genes *HLA-E* and *-G* ectopically in the *AAVS1* site, a safe harbor locus, in human cells via recombinase-mediated cassette exchange (RMCE) 23. A cassette including floxed *PGK* promoter-driven *puΔTK* was inserted into *AAVS1* in the *B2M^-/-^* hESCs to establish a master hESC line for RMCE (Fig. 2A). One single cell-derived clone was selected to verify the integration of the cassette into the *AAVS1* site. Polymerase chain reaction (PCR) results showed that the clone was heterozygous with the cassette integrated into one allele of the *AAVS1* site as the borders between the insert and its flanking regions were amplified but the full insert was too long (around 2.5 kb) to be amplified (Fig. 2B). Both HLA-Ia and -Ib require the subunit B2M for assembly and presence on the cell surface 24. Thus, we fused *B2M* with *HLA-E* or *-G* using a linker containing 4 repeats of gly-gly-gly-gly-ser, namely (G_4_S)_4_, to generate single-chain dimers (E-SCD or G-SCD) 25 (Fig. 2C), which were further linked with a neomycin-resistant gene *NeoR* via the P2A peptide 26, together forming an RMCE exchange donor vector for both *HLA-E* and *-G* expression (Fig. 2D).

Next, we replaced the *PGK* promoter-driven *puΔTK* in the cassette in the master hESCs with the donor vector through Cre recombinase-mediated RMCE (Fig. 2D). One single-cell clone was selected and verified via PCR for the successful exchange in the master hESC line based on the detection of the flanking sequences of the transgenes *HLA-E* and *-G*, hence named *B^-^E^+^G^+^* hESCs (Fig. 2E). Further, both HLA-E and -G were detected on the surface of *B^-^E^+^G^+^* hESCs compared to the original master cells, and IFN-γ priming did not affect the surface expression of both HLA-E and -G (Fig. 2F). The pluripotency of the cells was verified via flow cytometry (Fig. S3A) and teratoma formation (Fig. S3B). PCR analysis further assured no integration of either the RMCE (Fig. S3C) or RMCE exchange donor vectors (Fig. S3D) in any other region in the genome. Thus, we introduced a single copy of *HLA-E* and *-G* into the *AAVS1* site of *B2M^-/-^* hESCs successfully.

### Site-specifically expressed HLA-E, but not HLA-G, increases NK resistance of only IFN-γ-primed *B2M^-/-^* EMSCs

**Figure 3:**
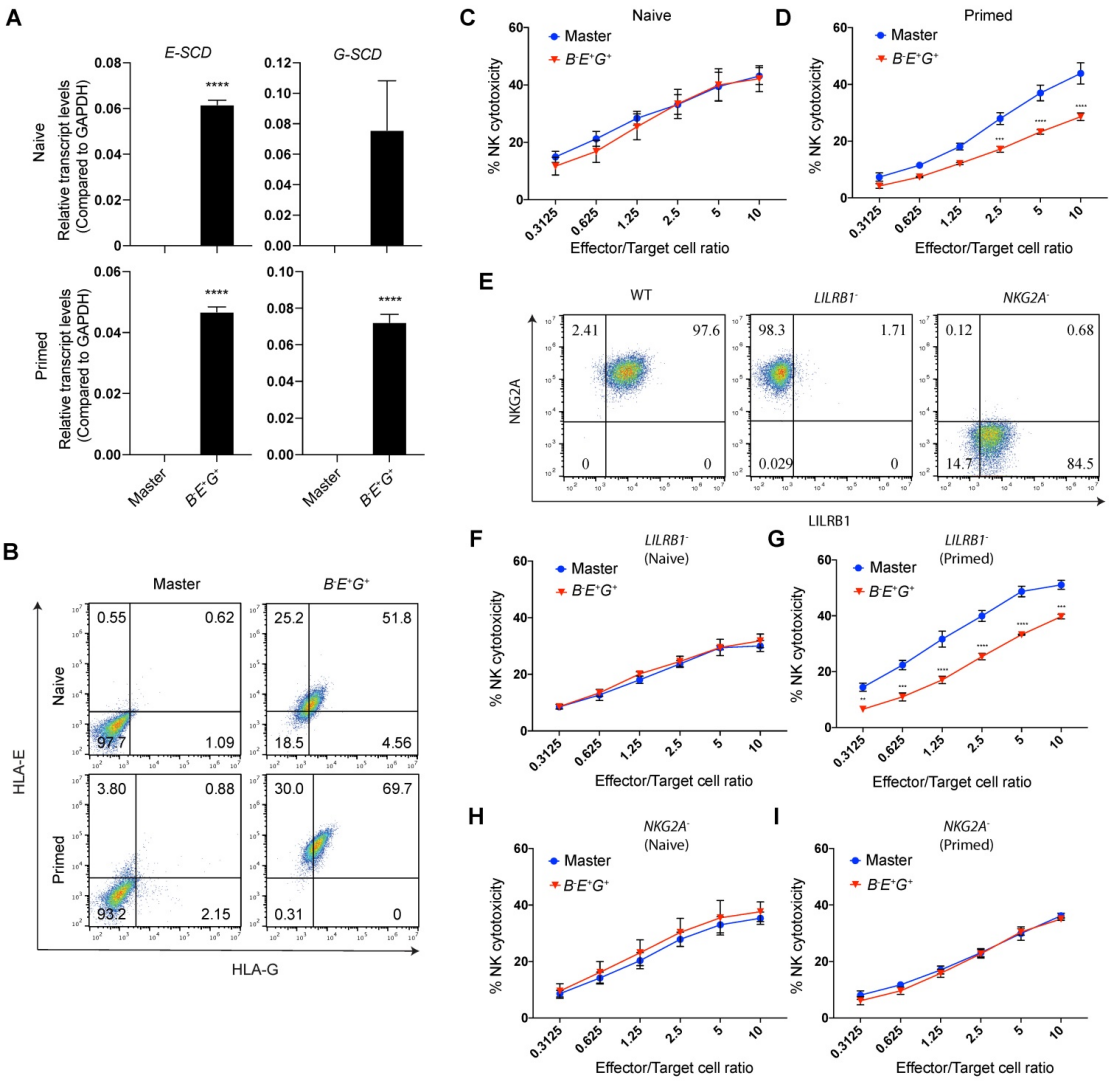
** Site-specifically expressed HLA-E, but not HLA-G, increases NK resistance of IFN-γ-primed *B2M^-/-^* EMSCs. (A)** qPCR analysis for *E-SCD* and* G-SCD* transcript levels in master and *B^-^E^+^G^+^* EMSCs with (primed) or without (naïve) IFN-γ treatment. N = 3, ^****^P < 0.0001 for *B^-^E^+^G^+^* versus master EMSCs per two-tailed unpaired *t* test. **(B)** Flow cytometry analysis for HLA-E and -G expression on the surface of master and *B^-^E^+^G^+^* EMSCs with or without IFN-γ treatment. **(C)** NK-92MI cell-mediated lysis of master and *B^-^E^+^G^+^* EMSCs (naïve). N = 3. **(D)** NK-92MI cell-mediated lysis of master and *B^-^E^+^G^+^* EMSCs (IFN-γ-primed). N = 3. ^**^P < 0.01, ^***^P < 0.001, ^****^P < 0.0001 for *B^-^E^+^G^+^* versus master EMSCs per two-way ANOVA followed by Sidak's multiple comparison test. **(E)** Flow cytometry analysis for NKG2A and LILRB1 expression on the surface of *LILRB1^-^* and *NKG2A^-^* NK-92MI cells. **(F)** *LILRB1^-^* NK-92MI cell-mediated lysis of master and *B^-^E^+^G^+^* EMSCs (naïve). N = 3. **(G)*** LILRB1^-^* NK-92MI cell-mediated lysis of master and *B^-^E^+^G^+^* EMSCs (IFN-γ-primed). N = 3, ^***^P < 0.001, ^****^P < 0.0001 for *B^-^E^+^G^+^* versus master EMSCs per two-way ANOVA followed by Sidak's multiple comparison test. **(H)** *NKG2A^-^* NK-92MI cell-mediated lysis of master and *B^-^E^+^G^+^* EMSCs (naïve). N = 3. **(I)** *NKG2A^-^* NK-92MI cell-mediated lysis of master and *B^-^E^+^G^+^* EMSCs (IFN-γ-primed). N = 3.

To test the NK resistance of the RMCE-derived *B^-^E^+^G^+^* hESCs, we differentiated them and the master hESCs as a control to MSCs, named *B^-^E^+^G^+^* and master EMSCs, respectively, which both expressed typical MSC markers at similar levels based on flow cytometry analysis (Fig. S4A) and retained the capability of trilineage differentiation *in vitro* (Fig. S4B). The expression levels of *E-SCD* and *G-SCD* in the *B^-^E^+^G^+^* EMSCs treated with or without IFN-γ were confirmed using quantitative PCR (qPCR) (Fig. 3A). Through flow cytometry, HLA-E and -G were detected on the surface of only the *B^-^E^+^G^+^* EMSCs but not the master EMSCs (Fig. 3B), suggesting successful generation of *B^-^E^+^G^+^* EMSCs. Interestingly, IFN-γ treatment increased the surface expression of both HLA-E and -G on the *B^-^E^+^G^+^* EMSCs (Fig. 3B), which might result from an increased supply of high-affinity immunopeptides for HLA-E and -G assembly after IFN-γ treatment 27, 28.

HLA-E and -G can inhibit NK cytotoxicity by binding to two major inhibitory receptors NKG2A/CD94 and LILRB1, respectively, on NK cells 11, 12. Thus, we tested whether *B^-^E^+^G^+^* EMSCs can escape NK killing by co-culturing them with NK-92MI cells. Unexpectedly, ectopic *HLA-E* and *-G* couldn't protect the cells from the NK cytotoxicity (Fig. 3C) unless they were primed with IFN-γ, in which around 20-50% NK inhibition was observed compared to the control master EMSCs (Fig. 3D). This result suggests that the site-specific expression of a single copy of *HLA-E* and *-G* is not sufficient to reduce the NK sensitivity of unprimed *B2M^-/-^* EMSCs, whereas IFN-γ priming assists the ectopic HLA-E or -G in executing the NK-inhibitory effect.

To test the contribution of *HLA-E* and *-G* in *B^-^E^+^G^+^* EMSCs to the NK inhibition, we generated *LILRB1^-^* and* NKG2A^-^* subtypes from NK-92MI cells using CRISPR/Cas9 (Figs. S5A) and confirmed the knockouts via flow cytometry (Fig. 3E). To examine the functionality of *LILRB1^-^* and *NKG2A^-^* NK-92MI cells, we tested their cytotoxicity against K562 cells negative for both HLA-E and -G 29. Compared to the WT control,* LILRB1^-^* and *NKG2A^-^* NK-92MI cells showed similar cytotoxicity against K562 cells (Fig. S5B), suggesting that knockout of *LILRB1* or* NKG2A* didn't compromise the general cytotoxic activity of the NK cell line. Interestingly, IFN-γ-induced NK inhibition only happened in *B^-^E^+^G^+^* EMSCs incubated with *LILRB1^-^* but not *NKG2A^-^* NK-92MI cells (Figs. 3F-I), suggesting the protective role of HLA-E in *B^-^E^+^G^+^* EMSCs after IFN-γ priming. Moreover, expression of genes involved in other NK-inhibitory mechanisms independent of B2M or HLA-Ia, including *PTGS2*, *IDO1*, *TGFB1*, *CD274*, *CD47*, and *PVR*, was similar between the master and *B^-^E^+^G^+^* EMSCs no matter whether primed with IFN-γ or not, although many of the genes were upregulated by IFN-γ priming in both master and *B^-^E^+^G^+^* EMSCs (Fig. S4C). This result implicates that the genetic manipulations didn't affect the expression of the irrelevant NK-regulatory genes.

### Lentivirus-transduced HLA-E and -G render dose-dependent NK resistance to *B2M^-/-^* EMSCs even without priming

**Figure 4:**
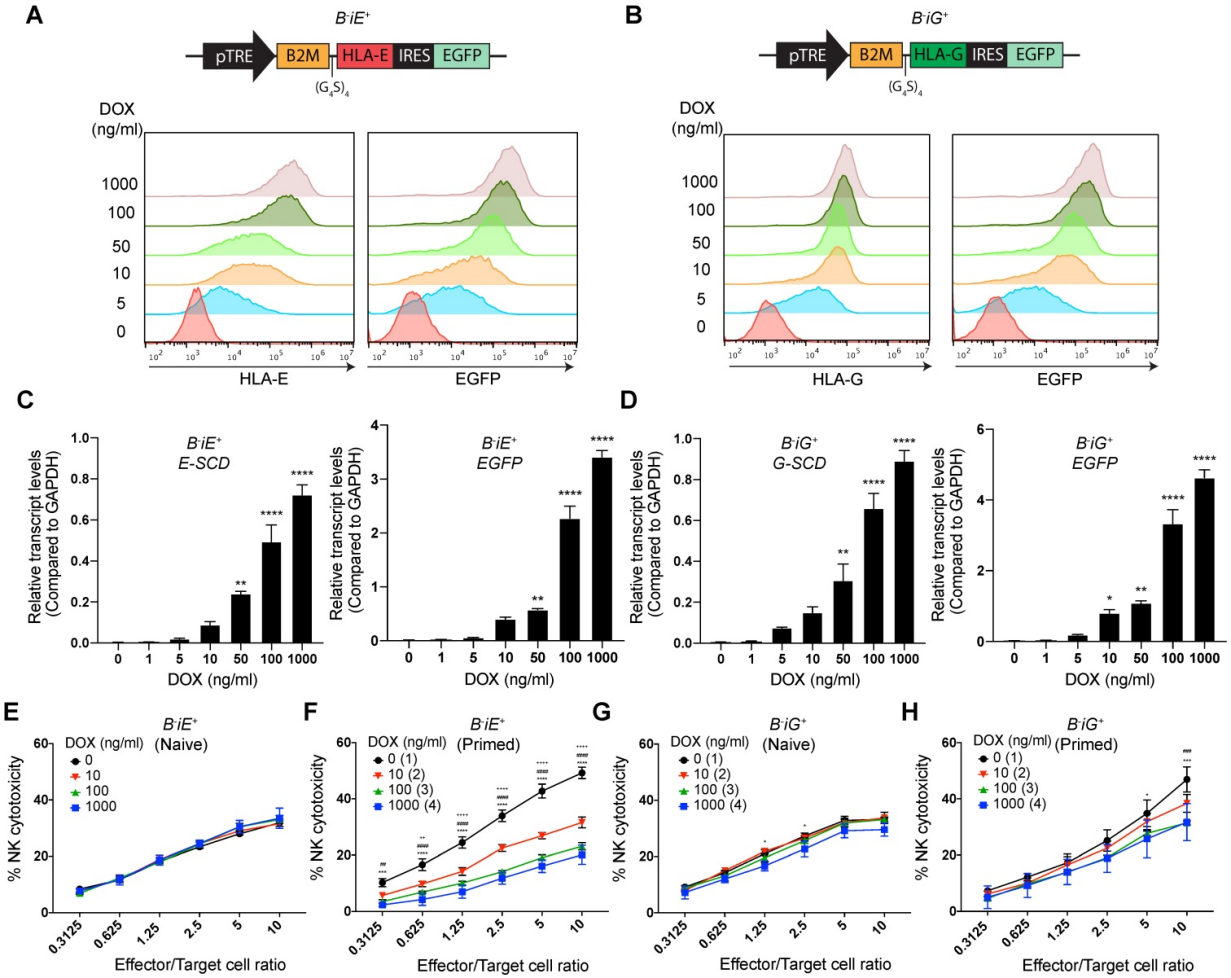
** HLA-E or -G inducibly expressed in *B2M^-/-^* EMSCs resist NK cytotoxicity dose-dependently. (A)** Schematic graph of the construct for inducible *E-SCD* expression and flow cytometry analysis for HLA-E and EGFP expression in *B^-^iE^+^* EMSCs treated with DOX. **(B)** Schematic graph of the construct for inducible *G-SCD* expression and flow cytometry analysis for HLA-G and EGFP expression in *B^-^iG^+^* EMSCs treated with DOX. **(C)** qPCR analysis of *E-SCD* and *EGFP* transcripts in *B^-^iE^+^* EMSCs under treatment with various concentrations of DOX. N = 3. ^**^P < 0.01 and ^****^P < 0.0001 for DOX-treated versus vehicle-treated *B^-^iE^+^* EMSCs per ordinary one-way ANOVA followed by Dunnette's multiple comparison test. **(D)** qPCR analysis of *G-SCD* and *EGFP* transcripts in *B^-^iG^+^* EMSCs under treatment with DOX. N = 3. ^*^P < 0.05, ^**^P < 0.01, and ^****^P < 0.0001 for DOX-treated versus vehicle-treated *B^-^iG^+^* EMSCs per ordinary one-way ANOVA followed by Dunnette's multiple comparison test. **(E)** NK-92MI cell-mediated lysis of *B^-^iE^+^* EMSCs (naïve) treated with DOX. N = 3. **(F)** NK-92MI cell-mediated lysis of *B^-^iE^+^* EMSCs (IFN-γ-primed) treated with DOX. N = 3. ^++^*P* < 0.01 and ^++++^*P* < 0.0001 for (2) versus (1); ^##^*P* < 0.01 and ^####^*P* < 0.0001 for (3) versus (1); ^***^P < 0.001 and ^****^P < 0.0001 for (4) versus (1) per two-way ANOVA followed by Dunnett's multiple comparison test. **(G)** NK-92MI cell-mediated lysis of *B^-^iG^+^* EMSCs (naïve) treated with DOX. N = 3. ^*^P < 0.05 for (4) versus (1) per two-way ANOVA followed by Dunnett's multiple comparison test. **(H)** NK-92MI cell-mediated lysis of *B^-^iG^+^* EMSCs (IFN-γ-primed) treated with DOX. N = 3. ^###^*P* < 0.001 for (3) versus (1); ^*^P < 0.05 and ^***^P < 0.001 for (4) versus (1) per two-way ANOVA followed by Dunnett's multiple comparison test.

Based on the results above, we wondered whether increased expression of *HLA-E* and *-G* can render such protection or not. To achieve gradient expression of both molecules, we employed a Tet-on based lentiviral vector to co-express enhanced green fluorescent protein (*EGFP*) and an *E-SCD* or *G-SCD* in the *B2M^-/-^* EMSCs under the control of DOX, named *B^-^iE*^+^ and *B^-^iG^+^* EMSCs, respectively (Figs. 4A and B). Flow cytometry and qPCR assays demonstrate DOX-induced expression of *HLA-E or E-SCD*, *HLA-G or G-SCD*, and *EGFP* in *B^-^iE*^+^ and *B^-^iG^+^* EMSCs were in a dose-dependent manner (Figs. 4A-D). Surprisingly, inhibited NK cytotoxicity was not observed in naïve *B^-^iE*^+^ and *B^-^iG^+^* EMSCs (Figs. 4E and G). Only after IFN-γ priming, the NK-inhibitory effects of the ectopic HLA-E and -G showed a clear parallel relationship with the DOX dose (Figs. 4F and H).

**Figure 5:**
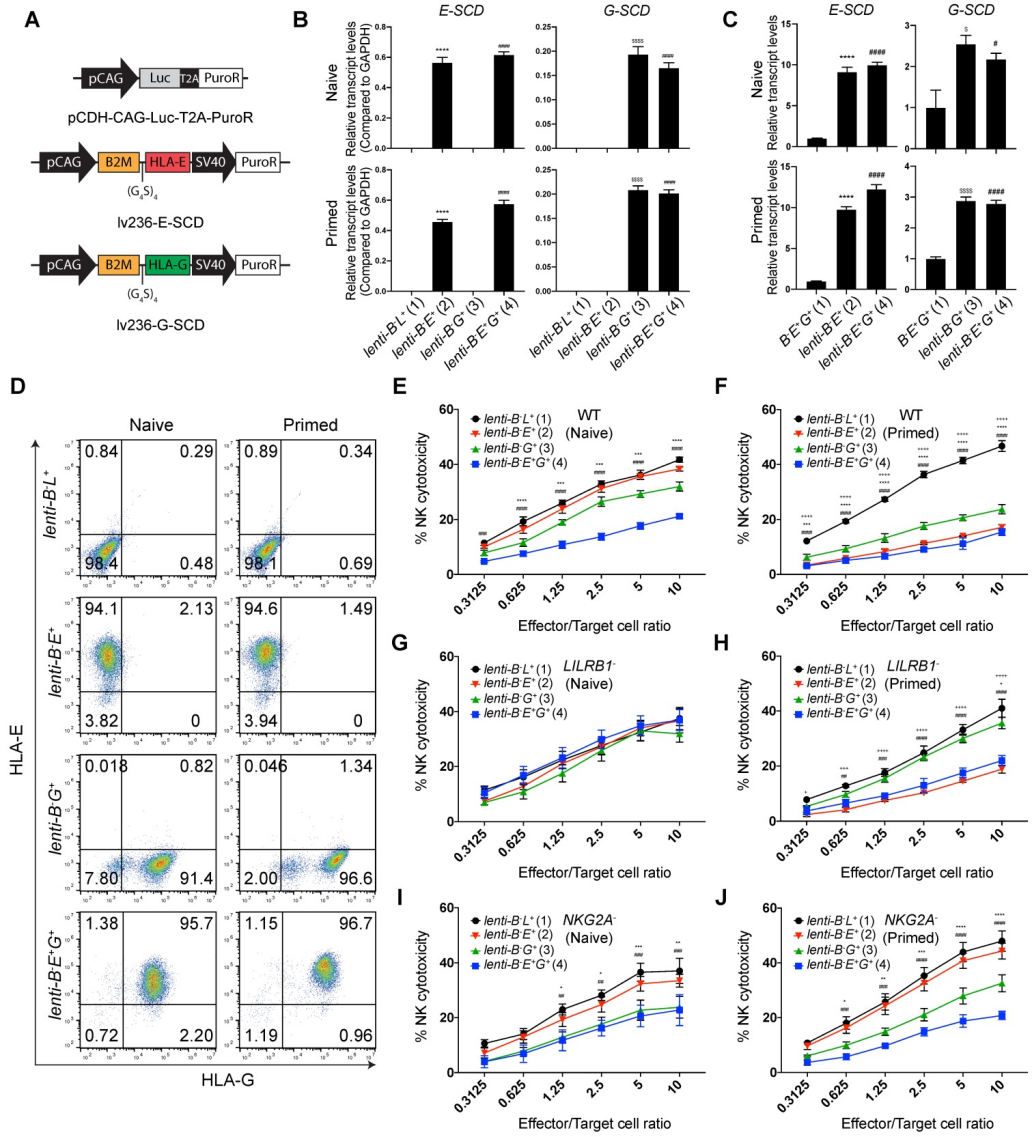
** EMSC ectopically expressing both HLA-E and -G resist NK cytotoxicity more than each alone. (A)** Schematic graphs of lentiviral transfer vectors overexpressing luciferase, E-SCD, and G-SCD, respectively. **(B)** qPCR analysis of *E-SCD and G-SCD* transcripts in *lenti-B^-^L^+^*, -*B^-^E^+^*, -*B^-^G^+^*, and -*B^-^E^+^G^+^* EMSCs. N = 3. ^****^P<0.0001 for (2) versus (1); ^$$$$^*P* < 0.0001 for (3) versus (1); ^####^*P* < 0.0001 for (4) versus (1) per ordinary one-way ANOVA followed by Dunnette's multiple comparison test. **(C)** qPCR analysis of relative transcript levels of *E-SCD or G-SCD* in *lenti*-*B^-^E^+^*, -*B^-^G^+^*, and -*B^-^E^+^G^+^* EMSCs compared to *B^-^E^+^G^+^* EMSCs. N = 3. ^****^P < 0.0001 for (2) versus (1); ^$^*P* < 0.05 and ^$$$$^*P* < 0.0001 for (3) versus (1); ^#^*P* < 0.05 and ^####^*P* < 0.0001 for (4) versus (1) per ordinary one-way ANOVA followed by Dunnette's multiple comparison test. **(D)** Flow cytometry analysis for HLA-E and -G expression on the surface of *lenti-B^-^L^+^*, -*B^-^E^+^*, -*B^-^G^+^*, and -*B^-^E^+^G^+^* EMSCs. **(E)** NK-92MI cell-mediated lysis of *lenti-B^-^L^+^*, -*B^-^E^+^*, -*B^-^G^+^*, and -*B^-^E^+^G^+^* EMSCs (naïve). N = 3, ^***^P < 0.001 and ^****^P < 0.0001 for (3) versus (1); ^###^*P* < 0.001 and ^####^*P* < 0.0001 for (4) versus (1) per two-way ANOVA followed by Dunnett's multiple comparison test. **(F)** NK-92MI cell-mediated lysis of *lenti-B^-^L^+^*, -*B^-^E^+^*, -*B^-^G^+^*, and -*B^-^E^+^G^+^* EMSCs (IFN-γ-primed). N = 3, ^++++^*P* < 0.0001 for (2) versus (1); ^***^P < 0.001, and ^****^P < 0.0001 for (3) versus (1); ^####^*P* < 0.0001 for (4) versus (1) per two-way ANOVA followed by Dunnett's multiple comparison test. **(G)** *LILRB1^-^* NK-92MI cell-mediated lysis of *lenti-B^-^L^+^*, -*B^-^E^+^*, -*B^-^G^+^*, and -*B^-^E^+^G^+^* EMSCs (naïve). N = 3. **(H)** *LILRB1^-^* NK-92MI cell-mediated lysis of *lenti-B^-^L^+^*, -*B^-^E^+^*, -*B^-^G^+^*, and -*B^-^E^+^G^+^* EMSCs (IFN-γ-primed). N = 3, ^+^*P* < 0.05, ^+++^*P* < 0.001, and ^++++^*P* < 0.0001 for (2) versus (1); ^*^P < 0.05 for (3) versus (1); ^##^*P* < 0.01, ^###^*P* < 0.001, and ^####^*P* < 0.0001 for (4) versus (1) per two-way ANOVA followed by Dunnett's multiple comparison test. **(I)** *NKG2A^-^* NK-92MI cell-mediated lysis of *lenti-B^-^L^+^*, -*B^-^E^+^*, -*B^-^G^+^*, and -*B^-^E^+^G^+^* EMSCs (naïve). N = 3, ^*^P < 0.05, ^**^P < 0.01, and ^***^P < 0.001 for (3) versus (1); ^##^*P* < 0.01, ^###^*P* < 0.001 for (4) versus (1) per two-way ANOVA followed by Dunnett's multiple comparison test. **(J)** *NKG2A^-^* NK-92MI cell-mediated lysis of *lenti-B^-^L^+^*, -*B^-^E^+^*, -*B^-^G^+^*, and -*B^-^E^+^G^+^* EMSCs (IFN-γ-primed). N = 3, ^*^P < 0.05, ^**^P < 0.01, ^***^P < 0.001, and ^****^P < 0.0001 for (3) versus (1); ^###^*P* < 0.001 and ^####^*P* < 0.0001 for (4) versus (1) per two-way ANOVA followed by Dunnett's multiple comparison test.

To achieve constitutive high-level expression of *HLA-E* and *-G*, we transduced *B2M^-/-^* EMSCs with a lentiviral vector containing a cassette for co-expression of a puromycin-resistant gene *PuroR* with *luciferase* (*Luc*) (as a control), *E-SCD* or *G-SCD* (Fig. 5A), resulting in *lenti-B^-^L^+^*, -*B^-^E^+^*, -*B^-^G^+^*, and -*B^-^E^+^G^+^* EMSCs, respectively, as confirmed via qPCR (Fig. 5B). The expression of *E-SCD* in the *lenti*-*B^-^E^+^* and *lenti*-*B^-^E^+^G^+^* EMSCs was 10-fold, and the expression of *G-SCD* in the *lenti*-*B^-^G^+^* and *lenti*-*B^-^E^+^G^+^* EMSCs was 2.5-fold, of that in the *B^-^E^+^G^+^* EMSCs generated via RMCE (Fig. 5C). The cell surface presence of HLA-E and -G was also confirmed using flow cytometry, which was further enhanced after IFN-γ priming (Fig. 5D).

To test the inhibitory effect of EMSCs on diverse NK subtypes, we used WT, *LILRB1^-^*, and *NKG2A^-^* NK-92MI cells to represent the three subtypes of primary NK cells:* NKG2A^+^*/*LILRB1^+^*, *NKG2A^+^*/*LILRB1^-^*, and *NKG2A^-^*/*LILRB1^+^*, respectively. The inhibitory effect on WT NK-92MI cells was observed the highest in *lenti*-*B^-^E^+^G^+^*, intermediate with *lenti*-*B^-^G^+^*, and the lowest with *lenti*-*B^-^E^+^*, compared to *lenti-B^-^L^+^* EMSCs control (Fig. 5E). Again, IFN-γ priming enhanced the inhibitory effect of *lenti*-*B^-^E^+^* and *lenti*-*B^-^G^+^*, while *lenti*-*B^-^E^+^G^+^* EMSCs still being the highest in NK inhibition (Fig. 5F). Consistently, *lenti*-*B^-^G^+^* and *lenti*-*B^-^E^+^* EMSCs lost resistance to *LILRB1^-^* and *NKG2A^-^* NK-92MI cells, respectively, and only *lenti*-*B^-^E^+^G^+^* EMSCs remained resistant to both NK subtypes especially following IFN-γ priming (Figs. 5G-J).

**Figure 6:**
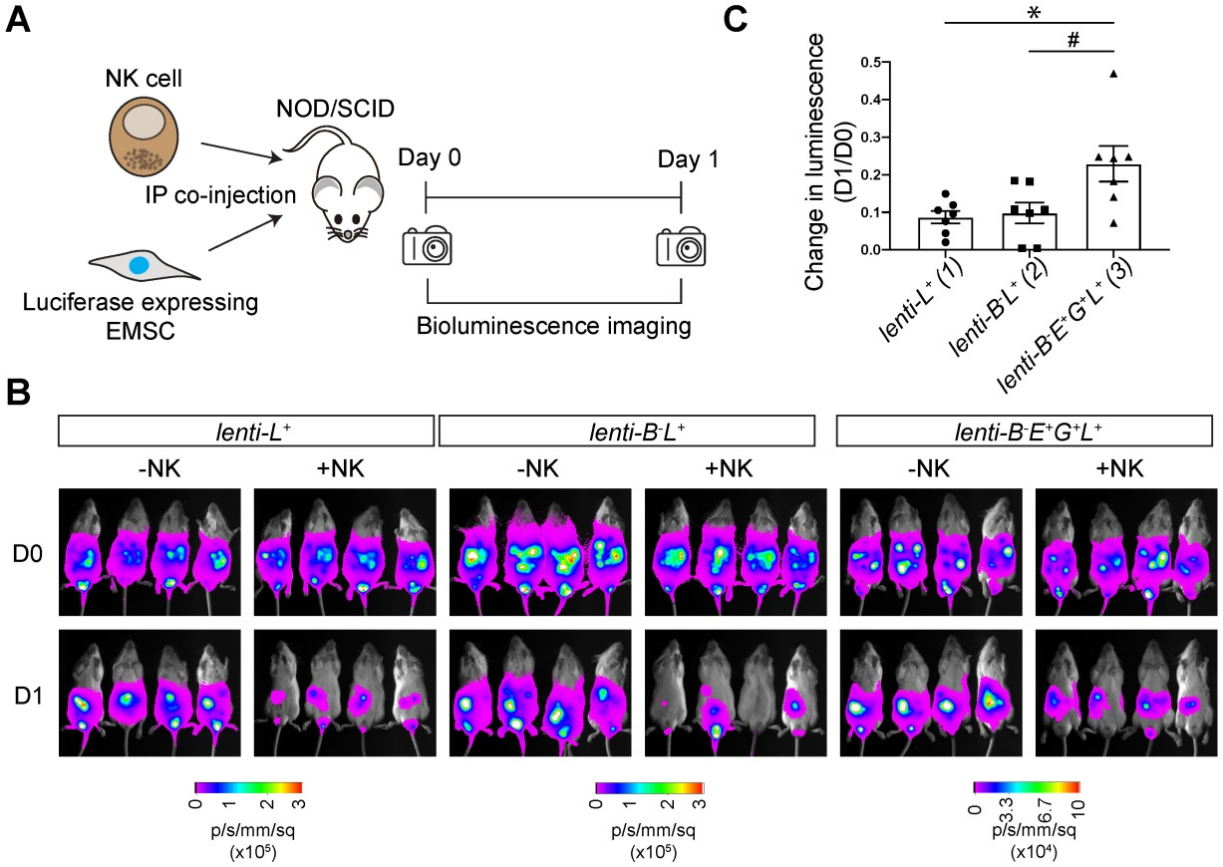
** HLA-E and -G protect *B2M^-/-^* EMSCs from NK cell-mediated lysis *in vivo*. (A)** Schematic graph of NK cell-mediated lysis of EMSCs *in vivo.*** (B)** *In vivo* luminescence changes after co-injection of NK-92MI cells with *lenti-L^+^*, *lenti-B^-^L^+^*, and *lenti*-*B^-^E^+^G^+^L^+^* EMSCs, N = 7. **(C)** Bioluminescence changes between day 0 (prior to NK-92MI cell injection) and day 1 after injection of NK-92MI cells with *lenti-L^+^*, *lenti-B^-^L^+^*, and *lenti*-*B^-^E^+^G^+^L^+^ EMSCs*, N = 7.^ *^P < 0.05 for (3) versus (1); ^#^*P* < 0.05 for (3) versus (2) per one-way ANOVA followed by Sidak's multiple comparison test.

To track transplanted cells *in vivo,* we transduced WT and *lenti*-*B^-^E^+^G^+^* EMSCs with a lentiviral vector containing a cassette for *EF1α* promoter-driven co-expression of *NeoR* linked with *luc,* resulting in* lenti-L^+^* and* lenti*-*B^-^E^+^G^+^L^+^* EMSCs, respectively. We tested NK resistance of the EMSCs *in vivo* by co-injecting NOD/SCID mice intraperitoneally (i.p.) with NK-92MI cells and *lenti-L^+^, lenti-B^-^L^+^,* or* lenti*-*B^-^E^+^G^+^L^+^* EMSCs (Fig. 6A). The bioluminescent signal in groups without NK-92MI cell co-injection was retained one day after the injection (Fig. 6B). Co-injection of NK-92MI cells reduced the bioluminescent signal, which was more obvious in mice injected with *lenti-L^+^* and* lenti-B^-^L^+^* EMSCs than in mice treated with *lenti*-*B^-^E^+^G^+^L^+^* EMSCs (Figs. 6B and C), suggesting that *lenti*-*B^-^E^+^G^+^L^+^* EMSCs resist NK cytotoxicity more than *lenti-L^+^* and* lenti-B^-^L^+^* EMSCs. Overall, these experiments demonstrate that a high-level of *HLA-E* and *-G* expression reduces NK cytotoxicity on *B2M^-/-^* EMSCs *in vivo*.

### Quality control of genetically manipulated EMSCs

First, we conducted Sanger sequencing of top 6 potential off-target sites of *B2M* sgRNAs in *lenti-L^+^* and *lenti-B^-^E^+^G^+^* EMSCs and found no mutation in any of the sites (Figs. S6A and B). Next, since genome editing often affects the expression of *TP53,* a tumor suppressor gene critical for maintaining genetic stability 30, 31, we tested its expression and found that both total and phosphorylated p53 levels were similar between *B2M^-/-^* and WT hESCs per western blotting following X-ray irradiation (Fig. S7), suggesting that *B2M* knockout didn't compromise *TP53* expression and function. Interestingly, total and phosphorylated p53 were absent in *B2M^-/-^* and WT EMSCs even after X-ray irradiation (Fig. S7), implying that *TP53* expression is repressed at this developmental stage.

To examine whether *B2M* knockout or lentiviral expression of *HLA-E* and *-G* affects the paracrine functions of EMSCs, we checked the cytokine profiles of *lenti-L^+^, lenti-B^-^L^+^*, and* lenti-B^-^E^+^G^+^* EMSCs. Surprisingly, we found that secretion of interleukin (IL)-6 and -8 reduced dramatically in *lenti-B^-^L^+^* EMSCs compared to *lenti-L^+^* EMSCs but recovered in *lenti-B^-^E^+^G^+^* EMSCs (Figs. S8A and B). This result indicates that B2M might assist the production of these cytokines in EMSCs through a not-yet-known mechanism, whereas overexpression of the B2M-fused HLA-E and -G can rescue the *B2M* knockout effect on the paracrine activity.

It has been reported that ectopic expression of either *HLA-E* or *-G* in tumor and virus-infected cells can evoke cytotoxic T lymphocyte (CTL) response 32, 33. To address this concern, we performed CTL assays and found that *lenti-B^-^E^+^G^+^* EMSCs didn't trigger the proliferation and activation of CD8^+^ T cells under both naïve and primed states compared to *lenti-L^+^* and* lenti-B^-^L^+^* EMSCs (Figs. S9A-D). These results suggest that ectopic expression of *HLA-E* and *-G* in EMSCs does not evoke CTL response, probably due to the immunosuppressive property of MSCs via secretion of PD-L1, PD-L2, CTLA-4, PGE2, IDO, and TGFβ1 to counteract T cell responses 34-39.

## Discussion

Although MSCs possess potent immunosuppressive capability by regulating both innate and adaptive immunity through cell-cell contact and secretion of soluble factors, they are not immune privileged as they, like other cell types, also elicit a humoral and cellular immune response *in vivo*. Following systemic infusion of human MSCs in immunocompromised mice, mouse MSCs in syngeneic mice, and rat MSCs in allogeneic rats, the majority of the cells die within 48 h after the transplantation 40. HLAs are the major factor to induce the T cell response 41, and depleting HLAs negates the T cell immunity but activates NK immunity 42. In this study, we found that EMSCs depleted of *B2M* became more sensitive to NK lysis than the WT control, and priming EMSCs with IFN-γ exacerbates the sensitivity. Site-specific knock-in of *HLA-E* and *-G* only showed a limited protective effect against NK cytotoxicity, whereas lentiviral transduction of *HLA-E* and *-G* dose-dependently reduced the NK sensitivity of *B2M^-/-^* EMSCs.

It has been reported that *B2M^-/-^* MSCs can escape the CTL-mediated killing *in vitro* 6-8. Moreover, *B2M^-/-^* allogeneic MSCs showed better therapeutic effects than WT MSCs on myocardial infarction and hindlimb ischemia partly due to escaping from CD8^+^ T cell-mediated immune rejection *in vivo* 7, 8. Nonetheless, *B2M^-/-^* MSCs exhibited increased NK sensitivity 6, and small interfering RNA (siRNA) against HLA class I also increased the susceptibility of MSCs to autologous NK cells 3. These results are consistent with our findings and can be explained by the “missing-self” effect of the HLA absence on the cell surface 42.

It has been known that IFN-γ treatment of MSCs inhibits the NK cytotoxicity against MSCs by increasing the expression of HLA class I and soluble factors, *e.g.*, PGE2 and IDO 3, 22. Unexpectedly, our results showed that IFN-γ treatment increased NK cytotoxicity to *B2M^-/-^* EMSCs, which might be explained by the increased expression of NK-activating genes such as *ICAM1*, *PVR*, and *Nectin 2* in MSCs after IFN-γ treatment 22 and the absence of the NK-inhibitory ligands HLA class I on the cell surface.

Both HLA-E and -G can protect allogeneic 14, 15 and xenogeneic cells 43-45 from NK cytotoxicity, respectively. Thus, Russel and coworkers reported that *HLA-E* knock-in protects *B2M^-/-^* hESC-derived CD45^+^ hematopoietic cells from primary NK-mediated lysis 14. However, we only observed such protection in IFN-γ-primed but not naïve *B2M^-/-^* EMSCs with site-specific insertion of *HLA-E* (Fig. 3). This result might result from differences between the present and previous studies in terms of the promoters (the *CAG* promoter versus the endogenous *B2M* promoter) used to drive *HLA-E* expression, the effectors (the NK-92MI cell line versus primary NK cells), and target cells (EMSCs versus hematopoietic cells). *HLA-G* knock-in into the *B2M* locus has also been reported to inhibit NK-92 cell-mediated cytotoxicity on cardiomyocytes differentiated from *B2M^-/-^* hESCs 16, which, however, failed to occur with *B2M^-/-^* EMSCs (Fig. 3). This might also be caused by reasons similar to the above, except that similar effector cells were used in both studies.

The major inhibitory receptors on NK cells for HLA-E and -G are NKG2A/CD94 and LILRB1 11, 12, respectively. *NKG2A^-^* and *LILRB1^-^* NK subpopulations vary in different individuals 17. We found that *B2M^-/-^* EMSCs with co-expression of *HLA-E* and *-G* showed robust resistance to *NKG2A^-^* and *LILRB1^-^* NK cells, while *HLA-E* and *-G* expression alone cannot provide resistance to *NKG2A^-^* and *LILRB1^-^* NK cells, respectively. This finding is consistent with a previous report that HLA-E provides limited protection to *B2M^-/-^* CD45^+^ cells against NK cells with low *NKG2A* expression 14. We have further clarified that HLA-G alone is insufficient for fully overcoming NK cytotoxicity, which is different from a previous study 15. Consistently, co-expression of *HLA-E* and *-G* protected *B2M^-/-^* EMSCs *in vivo* against WT NK-92MI cells representing *NKG2A^+^*/*LILRB1^+^* NK cells. Thus, co-expression of *HLA-E* and *-G* renders *B2M^-/-^* EMSCs resistant to broader NK subtypes, including *NKG2A^+^*/*LILRB1^+^*, *NKG2A^+^*/*LILRB1^-^*, and *NKG2A^-^*/*LILRB1^+^*, than expression of *HLA-E* or *-G* each alone. In addition, EMSCs express high levels of PD-L1 46 and CD47 (Fig. S4C), which may make EMSCs also resistant to *PD-1^+^*/*SIRPα^+^* NK cells 47, 48 and macrophages 49, 50, respectively.

Interestingly, naïve EMSCs with high-level expression of *HLA-E* presented no inhibitory effects on NK cells. In contrast, IFN-γ-primed EMSCs with a relatively low-level of *HLA-E* expression revealed significant inhibition against NK cell-mediated lysis. Peptides loaded by HLA-E have dramatic effects on the interaction of HLA-E with corresponding receptors. For example, HLA-E loaded with HLA-A2 peptide has more surface expression than HLA-E loaded with HLA-B7 peptide 27. HLA-E loaded with HLA-G peptide has higher affinity for CD94/NKG2A than HLA-E loaded with peptides from HLA-Ia 51. However, HLA-E loaded with heat shock protein 60 peptide loses the capability of interacting with NKG2A/CD94 and cannot inhibit *NKG2A^+^* NK cells 51. Based on these reports, IFN-γ-primed EMSCs might generate more high-affinity peptides loaded by HLA-E than naïve EMSCs.

It is worth noting that MSCs as well as other therapeutic cell types also encounter clearance in the host by the instant blood-mediated inflammatory reaction including coagulation, complement reaction, and phagocyte activation 52 and the lung or precapillary entrapment following systemic infusion 53-56. Furthermore, although HLA class II antigens are not expressed in naïve MSCs, they can be expressed in MSCs primed with cytokines like IFN-γ, undergoing hypoxia or following differentiation 57-61. Consistently, HLA class II molecules were detected on the surface of IFN-γ-primed, but not naïve, EMSCs, which were not affected by co-culture with NK cells. Interestingly, both *lenti-B^-^L^+^* and *lenti-B^-^E^+^G^+^* EMSCs had much higher expression of HLA class II on their surface than *lenti-L^+^* EMSCs (Figs. S10A and B). These results suggest that *HLA class II* expression might be regulated by multiple factors including B2M and upregulated following *B2M* knockout, which cannot be reversed by *HLA-E* and *-G* overexpression. Thus, it is also necessary to block the *HLA class II* expression in *B2M^-/-^* cells to generate fully immunoresistant hPSCs by knocking out genes such as *CIITA*, *RFXANK*, or *HLA class II* directly 62-64.

On the other hand, it is critical to evaluate other biological activities of B2M, CIITA, *etc*., and their loss of functions in targeted EMSCs and their progeny. Genetic manipulations are a double-edged sword as they can create novel biological functions of a cell and also damage the genomic integrity of the cell. We didn't detect any mutation in the top 6 potential off-target sites of *B2M* sgRNAs in *lenti-B^-^E^+^G^+^* EMSCs. Nevertheless, for future therapeutic application, whole-genome sequencing is required to identify any mutated genes and reduce the risk of tumorigenesis if an oncogene is triggered or a tumor-suppressor gene is disabled. To safeguard the use of hPSC progeny, a suicide gene such as iCaspase-9 is installed in the genome for quick clearance of targeted cells once they are becoming malignant or infected 65. Nevertheless, the ideal cells for therapy are those without any genomic manipulation.

In conclusion, this study demonstrates that ectopic expression of *HLA-E* and *-G* in *B2M^-/-^* EMSCs reverses enhanced NK sensitivity of the cells in a synergistic and dose-dependent manner. In addition, we suggest that dose-dependence should be considered when using HLA-E and -G to resist NK cytotoxicity. Moreover, IFN-γ priming increases, instead of, decreases NK sensitivity of *B2M^-/-^* EMSCs, whereas HLA-E and -G have more substantial protective effects on IFN-γ-primed *B2M^-/-^* EMSCs than naïve control cells, which may help the cells survive better in inflammatory diseases. The generation of NK-resistant MSCs from *B2M*^-/-^ hESCs shall greatly enhance their therapeutic efficacy via increased survival from both innate and adaptive immunity. More studies are warranted to evaluate the efficacy and biosafety of the cells in conditions with high diversity of NK cell populations in patients.

## Methods

### Cell culture

The experiments carried out in this project were in accordance with the National Institutes of Health Guidelines on Human Stem Cell Research. The protocols were approved by the University of Macau Panel of Research Ethics. H1 hESCs (Wicell, WA01) were maintained in monolayer on Matrigel (Corning, 354230) in E8 medium (Thermo, A1517001), 293T cells (ATCC, CRL-3216) in high glucose DMEM medium (Thermo, 11965092) with 10% FBS (Thermo, 16000044), K562 cells (ATCC, CCL-243) in RPMI1640 medium (Thermo, 11875093) with 10% FBS, NK-92MI cells (ATCC, CRL-2408) in αMEM (Thermo, 32561037) with inositol (Sigma, I5125, 0.2 mM), folic acid (Sigma, F8758, 0.02 mM), 2-mercaptoethanol (Thermo, 21985023, 0.1 mM), 12.5% horse serum (Thermo, 26050088) and 12.5% FBS, and MSCs in αMEM (Thermo, 12571063) with 20% FBS.

### MSC differentiation

MSC differentiation was performed as previously described 19, 20. In brief, hESCs were dissociated with TrypLE (Thermo, 12605010) and plated in the density of 90,000 cells/cm^2^ in E8 medium supplemented with ROCK inhibitor Y27632 (Stemgent, 04-0012, 10 μM) for 24 h. Cells were treated with Essential 6 (E6) medium (Thermo, A1516401) supplemented with sodium heparin (Sigma, H3149, 22.5 ng/ml), bFGF (Thermo, PHG0261, 10 ng/ml), CHIR99021 (Selleck, S1263, 1 μM), SB431542 (Stemgent, 04-0010, 10 μM) and dorsomorphin (R&D, 3093, 1 μM). Cells were split at a 1:6 ratio when they became confluent. Fifteen days later, cells were cultured in MSC medium for continued differentiation to MSCs for 5 more days. Following routine characterization for MSC markers and tri-lineage differentiation 66, the resulting cells were designated as EMSCs at passage 0 (p0). EMSCs within 10 passages were used in this study.

### Plasmid construction

#### Lentiviral transfer vectors for single-chain dimers of HLA-E (E-SCD) and HLA-G (G-SCD)

Firstly, B2M-linker was amplified from cDNA of H1 hESCs using primers B2M-cDNA-F/R and B2M-G4S-F/R carrying a (G_4_S)_4_ linker and Esp3I ends; HLA-E fragments were amplified from lv236-HLA-E (Genecopoeia) using primers HLA-E1-F/R and HLA-E2-F/R carrying Esp3I ends, and lv236-destination vector was constructed by inserting Esp3I cloning sites (annealed from Esp3I-anneal1-F/R) into KpnI- and NotI-digested lv236-mCherry (Genecopoeia). Then, B2M-linker, HLA-E fragments, and lv236-destination vector were assembled through Golden Gate Cloning 67. Similarly, HLA-G fragment with Esp3I ends was amplified from lv236-HLA-G1 using primers HLA-G-F/R. Then, B2M-linker, HLA-G fragment, and lv236-destination vector were assembled as above. The resultant lv236-E-SCD and lv236-G-SCD were verified using Sanger sequencing. Q5 DNA polymerase for PCR and restriction enzymes for DNA digestions were all from NEB for plasmid constructions.

#### RMCE donor vector

The left and right homologous arms (5' HA and 3' HA, respectively) amplified from Puro-Cas9 donor plasmid (Addgene # 58409, a gift of Danwei Huangfu) 68 were inserted into KpnI- or EcoRV-digested plasmid PGKdtabpA (Addgene #13440, a gift of Philippe Soriano) 69 to obtain PGK-HA-DTA-pA. In addition, the lox71-PGK-puΔTK-lox2272 fragment was digested from pLCA.66/2272 (Addgene #22733, a gift of Mark Magnuson) 70 and inserted into ClaI- and BstBI-digested PGK-HA-DTA-pA plasmid. The RMCE donor vector AAVS1-lox71-PGK-puΔTK-lox2272 was verified using Sanger sequencing.

#### E-SCD and G-SCD RMCE exchange vector

The E-SCD-P2A fragment with Esp3I ends was amplified from RMCE-E-SCD-P2A-NeoR (constructed elsewhere), the G-SCD fragment with Esp3I ends was amplified from the lv236-G-SCD, and the P2A-NeoR fragment with Esp3I ends was amplified from the pCDH-iC9-P2A-NeoR plasmid 65. The RMCE destination vector was constructed by inserting Esp3I sites into PacI- and BsrGI-digested RMCE-mCherry vector (constructed elsewhere). Then, the E-SCD-P2A, G-SCD, and P2A-NeoR fragments were assembled with the RMCE destination through Golden Gate Cloning. The RMCE exchange vector RMCE-E-SCD-G-SCD-NeoR was verified using Sanger sequencing.

#### Lentiviral transfer vectors for luciferase

The *luciferase* gene was amplified from the pGL3-control plasmid (Promega) and inserted into NotI- and EcoRI-digested pCDH-CAG-MSC-T2A-PuroR (Clontech) or AgeI- and BamHI-digested lenti-SpCas9 neo (Addgene #104996, a gift of Brett Stringer) 71 through Gibson assembly. The resultant pCDH-CAG-Luc-T2A-PuroR and plenti-EF1-Luc-P2A-NeoR were verified using Sanger sequencing.

#### Lentiviral transfer vector for inducible expression of E-SCD and G-SCD

E-SCD and G-SCD amplified from the lv236-E-SCD and lv236-G-SCD, respectively, were inserted into BamHI- and NdeI-digested pTight-TYR-EGFP plasmid (constructed elsewhere) through Gibson assembly. The resultant pTight-E-SCD-IRES-EGFP and pTight-G-SCD-IRES-EGFP were verified using Sanger sequencing.

### Lentiviral packaging and transduction

293T cells were transfected with lentiviral transfer vectors, pCMVR8.74 (Addgene #22036, a gift of Didier Trono), and pMD2.G (Addgene #12259, a gift of Didier Trono) at a ratio of 3:2:1 using the Lipofectamine 3000 transfection reagent (Thermo, L30000015). The supernatant containing lentiviral particles was collected 48 and 72 h after transfection. EMSCs were transduced with corresponding lentiviral particles supplemented with polybrene reagent (Sigma, TR1003, 10 μg/ml). 48 h later, cells were treated with the corresponding antibiotic puromycin (Sigma, P9620, 500 ng/ml) or G418 (Thermo, 10131027, 500 µg/ml) for two weeks to enrich transduced cells.

### Genome editing

#### *B2M* knockout

The guide sequences were designed according to the *B2M* gene using the online CRISPR design tool (crispr.mit.edu) as follows CGCGAGCACAGCTAAGGCCA and ACTCTCTCTTTCTGGCCTGG. Corresponding annealed oligos B2M-sg1-F/R and B2M-sg2-F/R (Table S1) were cloned into BbsI-digested pSpCas9n(BB)-2A-GFP (PX461) and pSpCas9n(BB)-2A-Puro (PX462) V2.0 (Addgene: #48140 and #62987, gifts of Feng Zhang) 72 to generate *B2M* targeting plasmids PX461-sgB2M1 and PX462-sgB2M2.

HESCs were transfected with *B2M* targeting plasmids using the Lipofectamine 3000 reagent. GFP^+^ cells were enriched using FACSAria III (BD Biosciences). Single-cell clones were obtained using the Incucyte Live Cell Analysis Imaging System (Sartorius). Two individual clones were selected, referred to as H1 *B2M^-/-^* #1 and #2, respectively. For genotyping, genomic DNA was extracted from both H1 *B2M^-/-^* #1 and #2 hESCs using the TIANamp Genomic DNA Extraction kit (Tiangen, DP304). Amplicons were obtained using Q5 DNA polymerase, cloned into T vectors, and verified via Sanger sequencing.

#### RMCE master line

H1 *B2M^-/-^* #2 hESCs were transfected with AAVS1-TALEN-L and AAVS1-TALEN-R (Addgene, gifts of Danwei Huangfu) 68 and the RMCE donor vector AAVS1-lox71-PGK-puΔTK-lox2272 using the Lipofectamine 3000 reagent. After puromycin selection, single-cell clones were obtained using the Incucyte system leading to the establishment of a RMCE master line. Site-specific integration of *puΔTK* in master hESCs was confirmed through PCR. Random integration was excluded through PCR.

#### *E-SCD* and *G-SCD* exchange

The master hESCs were transfected with pCAG-Cre:GFP (Addgene #13776, a gift of Connie Cepko) 73 and the exchange vector RMCE-E-SCD-G-SCD-NeoR (1:1) using the Lipofectamine 3000 reagent. After selection with G418, single-cell clones were obtained using the Incucyte system. Site-specific exchange of *E-SCD* and *G-SCD* in *B^-^E^+^G^+^* hESCs was confirmed through PCR. Random integration was excluded through PCR.

#### *LILRB1* and *NKG2A* knockout

The guide sequences were designed according to the sequences of *LILRB1* (gene id: 10859) and *NKG2A* (*KLRC1*, gene id: 3821) using the online CRISPR design tool as follows TGTACCACCACCTGCGACTC and AACAACTATCGTTACCACAG. Corresponding annealed oligos LILRB1-sg-F/R and NKG2A-sg-F/R (Table S1) were cloned into Esp3I-digested LentiCRISPRv2 (Addgene: #52961, a gift of Feng Zhang) 74. NK-92MI cells were transduced with the corresponding lentiviral particles. Puromycin-selected cells and WT controls were stained with anti-LILRB1 and -NKG2A antibodies (Table S2). *LILRB1^-^* and *NKG2A^-^* NK-92MI cells were enriched using FACSAria III.

### Teratoma assay

All animal procedures were conducted under the guidelines and animal use protocol (#UMARE-011-2019) approved by the University of Macau subpanel on animal research ethics. hESCs (~1 million) were suspended in 100 μl of cold Matrigel-DPBS (1:1) and injected subcutaneously into inguinal flanks of 4-6 weeks old NOD/SCID mice. Eight weeks after injection, mice were euthanized, and teratomas were dissected and analyzed using hematoxylin and eosin staining on the paraffin-embedded sections.

### Karyotyping

Cells were treated with KaryoMAX colcemid (Thermo, 15212012), followed by 25% acetic acid and 75% methanol fixation. The fixed cells were sent to Global Medical Laboratory Center (Wuhan, China) for G-band karyotyping.

### Western blotting

Cells were lysed in the radioimmunoprecipitation assay (RIPA) buffer (Thermo, 89900) supplemented with proteinase inhibitor (Thermo, 87786). 50 μg proteins per sample per lane were separated on a SDS-PAGE gel and then transferred to polyvinylidene difluoride (PVDF) membrane (Bio-Rad, 1620177). Membranes were blocked with 5% milk in phosphate-buffered saline-0.1% Tween 20 (Sigma, P7949) and then incubated with primary antibodies overnight at 4 °C, followed by corresponding secondary antibodies for 1 h at room temperature (Table S2). Detected signals were visualized with enhanced chemiluminescence and imaged using ChemiDoc Imager (Bio-Rad).

### Flow cytometry

Cells were harvested using TrypLE to obtain single-cell suspensions. For cell surface staining, cells were incubated with primary antibodies for 30 min on ice, followed by corresponding secondary antibodies for another 30 min on ice. For intracellular staining, cells were fixed with 4% paraformaldehyde for 15 min and permeabilized with 90% ice-cold methanol for 1 h or overnight at -20 °C. Cells were then blocked with 3% bovine serum albumin (Beyotime, ST023) for 1 h at room temperature, followed by incubation with primary antibodies for 1 h on ice and corresponding secondary antibodies for another 1 h on ice. Data were collected using the Cytoflex cytometer (Beckman Coulter) and analyzed using the FlowJo software (Treestar). Antibody information was listed in Table S2.

### Real-time qPCR

Total RNA was extracted using Trizol reagent (Thermo, 15596026) according to the manufacturer's instructions. Reverse transcription was performed using the PrimeScript RT Reagent Kit with gDNA Eraser (Takara, RR047A). qPCR was carried out using iTaq Universal SYBR Green Supermix (Bio-rad, 1725124) with gene-specific primers (Table S3) in CFX96 Touch Real-Time PCR Detection System (Bio-Rad). Gene expression levels were normalized to that of GAPDH.

### Trilineage differentiation

MSCs were differentiated into adipocytes, osteoblasts, and chondrocytes using the corresponding StemPro differentiation medium (Thermo, A1007001, A1007201, and A1007101). In brief, cells were seeded into 24-well plates and cultured in the corresponding differentiation medium. For adipogenesis, cells were fixed and stained with Oil Red O (Sigma, O0625) at 40 days after differentiation. For osteogenesis and chondrogenesis, cells were fixed and stained with Alizarin Red S (Sigma, A5533) and Alcian Blue (Sigma, 109-09), respectively, at 21 days after differentiation. Staining was visualized under a microscope (Axio observer, Carl Zeiss).

### NK cytotoxicity assay *in vitro*

Ten thousand Calcein-AM (ebioscience, 65-0853-78, 5 µM)-labeled target cells (MSCs or K562 cells) were seeded in 100 μl per well in a flat-bottom 96-well plate. Effector cells (NK-92MI) were added in 100 μl at different effector to target cell ratios and incubated for 4 h at 37 °C. All groups were set up in triplicate. Target cells without NK cells were used for spontaneous release and target cells lysed with 1% Triton X-100 (Sigma, T8787) for maximum release. 100 μl supernatant was collected and measured using Victor X5 microplate reader (PerkinElmer) with excitation filter at 485 nm and emission filter at 535 nm. Percent lysis was calculated using the following formula:



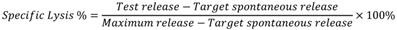



### NK cytotoxicity assay *in vivo*

NOD/SCID mice were injected i.p. with 2.5×10^5^ luciferase-expressing EMSCs. Four hours after EMSCs injection, 2.5×10^6^ NK-92MI cells were injected i.p. per mouse. Bioluminescence imaging was performed using In-Vivo Xtreme imaging system (Bruker) at days 0 and 1 for pre-NK-92MI and post-NK-92MI luciferase signals, respectively. The average change in the luminescence of NK-92MI non-injected mice was used as control.

### Statistics

Statistical analysis was carried out with Prism 9 software (GraphPad). All data shown in the study are presented as mean ± S.E.M. Statistically significant results were considered when P < 0.05. Two-tailed, unpaired *t* tests, ordinary one-way ANOVA tests, and two-way ANOVA tests were used to analyze the data in this study.

## Supplementary Material

Supplementary methods, figures and tables.

## Abbreviations

MSC: mesenchymal stem cell
HLA: human leukocyte antigen
HLA-Ia: classical HLA class I
iPSC: induced pluripotent stem cell
WT: wild-type
KIR: killer-cell immunoglobulin-like receptor
NK: natural killer
HLA-Ib: non-classical HLA class I
NKG2A: natural killer group 2, member A
LILRB1: leukocyte immunoglobulin-like receptor subfamily B member 1
hPSC: human pluripotent stem cell
ESC: embryonic stem cell
hESC: human embryonic stem cell
B2M: β-2-microglobulin
EMSC: hESC-derived MSC
DOX: doxycycline
CRISPR: clustered regularly interspaced short palindromic repeats
Cas9: CRISPR-associated protein 9
IFN-γ: interferon-gamma
RMCE: recombinase-mediated cassette exchange
PCR: polymerase chain reaction
E-SCD: HLA-E single-chain dimer
G-SCD: HLA-G single-chain dimer
qPCR: quantitative PCR
PTGS2: prostaglandin-endoperoxide synthase 2
IDO: indoleamine 2,3-dioxygenase
TGFβ1: transforming growth factor beta 1
PVR: poliovirus receptor
EGFP: enhanced green fluorescent protein
Luc: luciferase
i.p.: intraperitoneally
IL: interleukin
CTL: cytotoxic T lymphocyte
PD-L1/2: programmed death-ligand 1/2
CTLA-4: cytotoxic T-lymphocyte-associated protein 4
PGE2: prostaglandin E2
siRNA: small interfering RNA
ICAM1: intercellular adhesion molecule 1
PD-1: programmed cell death protein 1
SIRPα: signal-regulatory protein alpha
CIITA: MHC class II transactivator
RFXANK: regulatory factor x associated ankyrin containing protein
