# Supplementary material for: Engineering of human mesenchymal stem cells resistant to multiple natural killer subtypes
Source: Int J Biol Sci. 2022 Jan 1;18(1):426–40. doi: 10.7150/ijbs.64640 (PMC8692142; doi:10.7150/ijbs.64640)
Supplement: Supplementary file 1 — Supplementary methods, figures and tables. [file ijbsv18p0426s1.pdf]

Supplemental Information

Engineering of human mesenchymal stem cells resistant to multiple natural killer subtypes

Dejin Zheng, Xiaoyan Wang, Zhenwu Zhang, Enqin Li, Cheungkwan Yeung, Roma Borkar, Guihui Qin, Yaojiong Wu, and Ren-He Xu

Supplemental Methods

Human cytokine antibody array

500,000 EMSCs were plated into each well of a 6-well plate and cultured in MSC complete medium (α-MEM plus 20% FBS) for 48 h. Conditioned medium was collected and analyzed using the Human Cytokine Antibody Array C5 kit (RayBiotech, AAH-CYT-5-8) according to the manufacturer's instructions. Fresh MSC complete medium was used as a negative control.

$CD8^+$ T cell proliferation assay

EMSCs were used as stimulator cells. Peripheral blood mononuclear cells (PBMCs) labelled with carboxyfluorescein succinimidyl ester (CFSE) (AAT Bioquest, 22022) at 2 μM were used as effector cells. 100,000 naïve or IFN-γ-primed EMSCs were used to co-incubate with 500,000 CFSE-labeled PBMCs in RPMI1640 medium supplemented with 10% FBS for 5 days in 24-well plates. PBMC were then stained with CD8-APC antibody (Table S2). Data were collected using the Cytoflex cytometer and analyzed using the FlowJo software.

PBMCs cultured for 5 days without target cells served as a negative control. Cells activated with phytohemagglutinin (PHA) (Sigma, 61764) at 5 ng/ml were used as a positive control.

CD8^{+} T cell activation assay

EMSCs were used as stimulator cells and PBMCs as effecter cells. Co-culture of effector and target cells was similar to above. After a 3-day co-culture, cells were stained with CD69-FITC and CD8-APC antibodies (Table S2). Data were collected using the Cytoflex cytometer and analyzed using the FlowJo software. PBMCs cultured for 3 days without target cells served as a negative control and PHA-activated cells as a positive control.

Off-target screening

Top 6 potential off-target sites of *B2M* sgRNAs were predicted using the online software Cas-OFFinder (www.rgenome.net/cas-offinder/) and amplified from genomic DNA extracted from WT and genetically edited EMSCs using corresponding primers (Table S1). PCR products were sequenced to identify mutations in the predicted sites.

Supplemental Tables

Table S1. List of oligos and primers used for plasmid construction in this study

**Primer name**	**Sequences (5'-3')**
B2M-sg1-F	CACCGCGCGAGCACAGCTAAGGCCA
B2M-sg1-R	AAACTGGCCTTAGCTGTGCTCGCGC
B2M-sg2-F	CACCGACTCTCTCTTTCTGGCCTGG
B2M-sg2-R	AAACCCAGGCCAGAAAGAGAGAGTC
B2M-T7-F	GGGAGGAACTTCTTGGCACA
B2M-T7-R	GACGCTTATCGACGCCCTAA
NKG2A-sg-F	CACCGAACAACTATCGTTACCACAG
NKG2A-sg-R	AAACCTGTGGTAACGATAGTTGTTC
LILRB1-sg-F	CACCGTGTACCACCACCTGCGACTC
LILRB1-sg-R	AAACGAGTCGCAGGTGGTGGTACAC
B2M-cDNA-F	CCGAGATGTCTCGCTCCGTG
B2M-cDNA-R	TGCTTACATGTCTCGATCCCAC
HLA-E1-F	ACGTCTCAGATCCGGCTCCCACTCCTTGAAGTATTT
HLA-E1-R	ACGTCTCATTTCCTTCCCCTTCTCCAGGTAT
HLA-E2-F	ACGTCTCTGAAACGCTGCTTCACCTGGAG
HLA-E2-R	ACGTCTCTAAACGCTACAAGCTGTGAGACTCAG
B2M-G4S-F	ACGTCTCACACCATGTCTCGCTCCGTGGCCTTAG
B2M-G4S-R	ACGTCTCGGATCCTCCTCCTCCAGAACCACCACCACCAGATC CTCCTCCTCCAGAACCACCACCACCCATGTCTCGATCCCACTT AACTAT

Esp3I-anneal1-F	GCCACCGGAGACGGACGTCTCTGTTTA
Esp3I-anneal1-R	GGCCTAAACAGAGACGTCCGTCTCCGGTGGCGTAC
HLA-G-F	ACGTCTCGGATCCGGCTCCCACTCCATGAGGTATTTC
HLA-G-R	ACGTCTCTAAACGCTAATCTGAGCTCTTCTTTCT
Luc1-F	AAAAGAATTCGCCGCCACCATGGAAGACGCC
Luc1-R	TTTTGCGGCCGCCACGGCGATCTTTCCGCC
Luc2-F	CGCCAGAACACAGGACCGGTGCCACCATGGAAGACGCC
Luc2-R	AAGTTTGTTGCGCCGGATCCCACGGCGATCTTTCCGC
AAVS1-LHA-F	AGGGAACAAAAGCTGCTTTGCTTTCTCTGACCAGCATTCTCT C
AAVS1-LHA-R	TCGAGGGGGGCCCGGCCCCACTGTGGGGT
AAVS1-RHA-F	ATCGATAAGCTTGATTTCGAAACTAGGGACAGGATTGGTGAC AG
AAVS1-RHA-F	CCGGTAGAATTCGATAGAGCAGAGCCAGGAACCC
Esp3I-anneal2-F	GCCACCGGAGACGGACGTCTCTGTCAA
Esp3I-anneal2-F	GTACTTGACAGAGACGTCCGTCTCCGGTGGCAT
P2A-R	ACGTCTCAAGGTCCAGGGTTCTCCTCCAC
G-SCD-F	ACGTCTCAACCTATGTCTCGCTCCGTGGCCTTAG
G-SCD-R	ACGTCTCATTCCATCTGAGCTCTTCTTTCTCCACAGC
P2A-F	ACGTCTCAGGAAGCGGAGCTACTAACTTCAGCCTGCTGAAGC AGGCTGGCGACGTGGAGGAGAACCCTGGACCTATGCTTGAG GGAGTGCAGGT
P2A-R1	ACGTCTCGTGACGTCAGAAGAACTCGTCAAGAAG

iEG-SCD-F	TGAACCGTCAGATCGCCTGGAGAAGCCACCATGTCTCGCTCCGTGGCCTTAG
iE-SCD-R	GGGAGAGGGGCGGATCGTTTAAACAGCTACAAGCTGTGAGACTCAG
iG-SCD-R	GGGAGAGGGGCGGATCGTTTAAACAGCTAATCTGAGCTCTTCTTTCT
F1	AACTCTGCCCTCTAACGCTG
R1	AGGGGAGGAGTAGAAGGTGG
F2	TCTATGGCTTCTGAGGCGGA
R2	GGTCCAGGCCAAGTAGGTG
F3	TGCCATCTCTCGTTTCTTAGGATG
R3	CGTACTTGGCATATGATACACTTGATG
F4	GATCTCCTGTCATCTCACCTTGCT
R4	GGGTTAGACCCAATATCAGGAGAC
F5	CTGGGACATATTCCTCCGCC
R5	ACATAACCAGGTTTAGTCCCGT
F6	CCTCGACTGTGCCTTCTAGT
R6	CGATTTAGAGCTTGACGGGGA
Off-target 1-F	CTCGTTGTACAGATGTGGTCACT
Off-target 1-R	AAGATACCCAGAGGGTACTGGTC
Off-target 2-F	GAAACAGCAGGAAACACACTAGG
Off-target 2-R	TCTCACGACTCCTCATGGATTTG
Off-target 3-F	CCTCCTAGTGTTGCTTGGGATAC
Off-target 3-R	CTCAACTGCATTTGTACGGTCTT

Off-target 4-F	AACCACTTGGGAGGACCTAAATC
Off-target 4-R	GACCGTCGATGTTAAAAAGCACA
Off-target 5-F	ATAACTACAGCTTCCTCCGTGTG
Off-target 5-R	ACACTCACCTTTGAGCATCTTGA
Off-target 6-F	GAGGTATTGAGGGGTTTCCACAT
Off-target 6-R	CCAGAGAGATTTAGTCCCCACAG

Table S2. Antibodies used in this study

Antibody	Host	Catalog No.	Dilution	Vender
B2M	Mouse	SAB4700010	1:1000 (FC)	Sigma
B2M	Rabbit	AB75853	1:1000 (WB)	Abcam
HLA-ABC-FITC	Mouse	11-9983-42	5 µl/test (FC)	eBioscience
HLA-ABC	Mouse	AB15680	1:1000 (WB)	Abcam
HLA-DR, DP, DQ	Mouse	555557	1:1000 (FC)	BD Biosciences
HLA-E	Mouse	14-9953-82	0.5ug/test (FC)	eBioscience
HLA-G-PE	Mouse	ab24384	5 µl/test (FC)	Abcam
MSC analysis kit	Mouse	562245	5µl/reaction (FC)	BD Biosciences
CD107a-FITC	Mouse	555800	5 µl/test (FC)	BD Biosciences
TRA-1-60	Mouse	16288	1:100 (FC)	Abcam
NANOG	Mouse	173368	1:100 (FC)	Abcam
OCT4	Rabbit	PA5-27438	1:100 (FC)	Thermo
OCT4	Mouse	SC-365509	1:100 (FC)	Santa Cruz
P53	Mouse	SC-98	1:1000 (WB)	Santa Cruz
p-P53 (S15)	Mouse	9286S	1:1000 (WB)	Cell Signaling
GAPDH	Mouse	SC-69778	1:1000 (WB)	Santa Cruz
NKG2A-APC	Mouse	A60797	5 µl/reaction (FC)	Beckman Coulter
LILRB1-PE	Mouse	16014-MM06-P	5 µl/reaction (FC)	SinoBiological

CD8-APC	Mouse	10980-MM28-A	5 µl/reaction (FC)	SinoBiological
CD69-FITC	Mouse	11150-MM06-F	5 µl/reaction (FC)	SinoBiological
FITC Isotype Control	Mouse	555742	20 µl/reaction (FC)	BD Biosciences
PE Isotype Control	Mouse	559320	20 µl/reaction (FC)	BD Biosciences
APC Isotype Control	Mouse	555751	20 µl/reaction (FC)	BD Biosciences
Alexa Fluor 488, anti-Mouse IgG	Donkey	A21202	1:1000 (FC)	Invitrogen
Alexa Fluor 647, anti-Mouse IgG	Donkey	A31571	1:1000 (FC)	Invitrogen
Alexa Fluor 647, anti-Rabbit IgG	Donkey	A31573	1:1000 (FC)	Invitrogen
StrepTactin-HRP Conjugate	-	1610381	1:20000 (WB)	Bio-rad
HRP, anti-Rabbit IgG	Goat	G-21234	1:10000 (WB)	Invitrogen
HRP, anti-Mouse IgG	Goat	G-20140	1:10000 (WB)	Invitrogen

Table S3. qPCR primers used in this study

**Gene**	**Sequence (5'-3')**
E-SCD	AGTATGCCTGCCGTGTGAAC
	GGGACACGGAAGTGTGGAAATA
G-SCD	CACAGCCCAAGATAGTTAAGTGG
	GGCGGCGCTGAAATACCTC
PTGS2	ATGCTGACTATGGCTACAAAAGC
	TCGGGCAATCATCAGGCAC
IDO1	GCCAGCTTCGAGAAAGAGTTG
	ATCCCAGAACTAGACGTGCAA
TGFB1	GGCCAGATCCTGTCCAAGC
	GTGGGTTTCCACCATTAGCAC
CD274	GCTGCACTAATTGTCTATTGGGA
	AATTCGCTTGTAGTCGGCACC
CD47	AGAAGGTGAAACGATCATCGAGC
	CTCATCCATACCACCGGATCT
PVR	TGGAGGTGACGCATGTGTC
	GTTTGGACTCCGAATAGCTGG
EGFP	TCGTGACCACCCTGACCTA
	GGTCTTGTAGTTGCCGTCG
GAPDH	AGGGCTGCTTTTAACTCTGGT
	CCCCACTTGATTTTGGAGGGA

Supplemental Figures

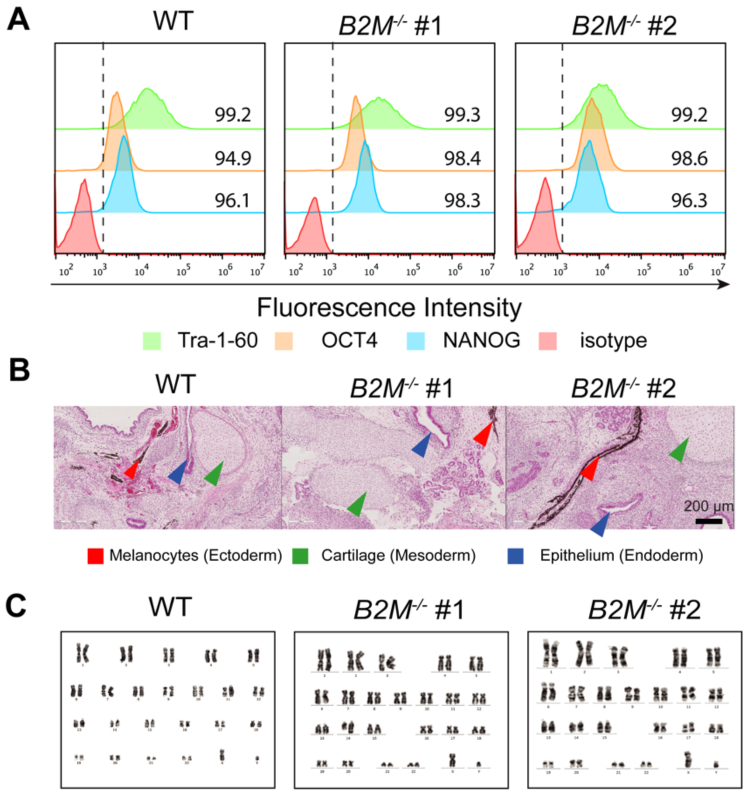

Figure S1. Pluripotency and karyotypes of *B2M*$^{-/-}$ hESCs.

(A) Flow cytometry analysis of WT and *B2M*$^{-/-}$ clone #1 and 2 hESCs for pluripotent markers including Tra-1-60, OCT4, and NANOG.

(B) Histological analysis of teratomas derived from WT and *B2M*$^{-/-}$ hESCs. Scale bar, 200 µm, N = 5.

(C) Karyotyping analysis of WT and *B2M*$^{-/-}$ hESCs.

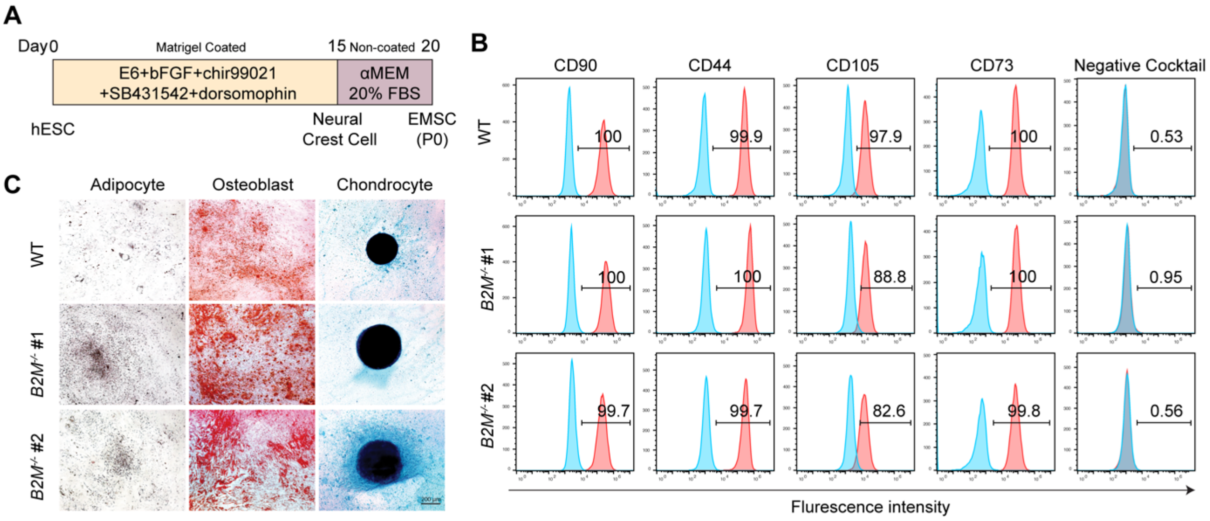

Figure S2. Characterization of *B2M*$^{-/-}$ EMSCs.

(A) Schematic graph of the protocol for hESC differentiation to MSC (EMSC).

(B) Flow cytometry analysis of WT and *B2M*$^{-/-}$ EMSCs for MSC markers including CD90, CD44, CD105, and CD73 and a negative marker cocktail.

(C) Trilineage differentiation from WT and *B2M*$^{-/-}$ EMSCs. Scale bar, 200 μm.

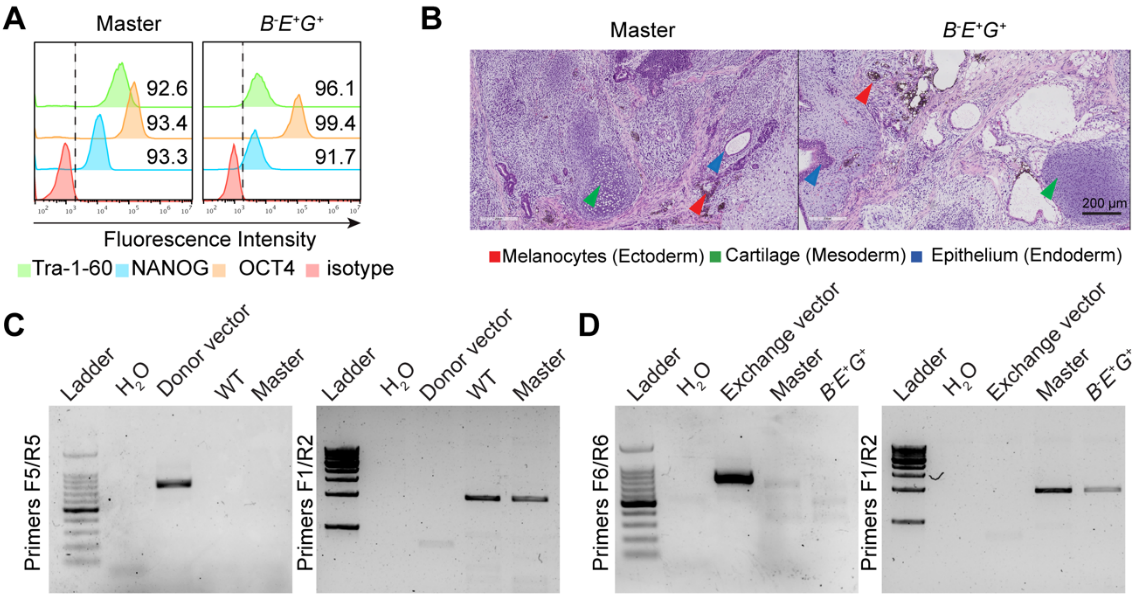

Figure S3. Quality control of master and $B^-E^+G^+$ hESCs.

(A) Flow cytometry analysis of master and $B^-E^+G^+$ hESCs for pluripotent markers Tra-1-60, OCT4, and NANOG.

(B) Histological analysis of teratomas formed by master and $B^-E^+G^+$ hESCs. Scale bar, 200 μm.

(C) PCR for detection of random integration of the RMCE donor vector into the genome of master hESCs. PCR products were amplified from H_2O, donor vector, and genomic DNA from WT and master hESCs using primes F5/R5 (left) and F1/R2 (right, a loading control) as indicated in Fig. 2A.

(D) PCR for detection of random integration of the exchange vector into the genome of $B^-E^+G^+$ hESCs. PCR products were amplified from H_2O, exchange vector, genomic DNA from master and $B^-E^+G^+$ hESCs using primes F6/R6 (left) and F1/R2 (right, a loading control) as indicated in Fig. 2A and 2D.

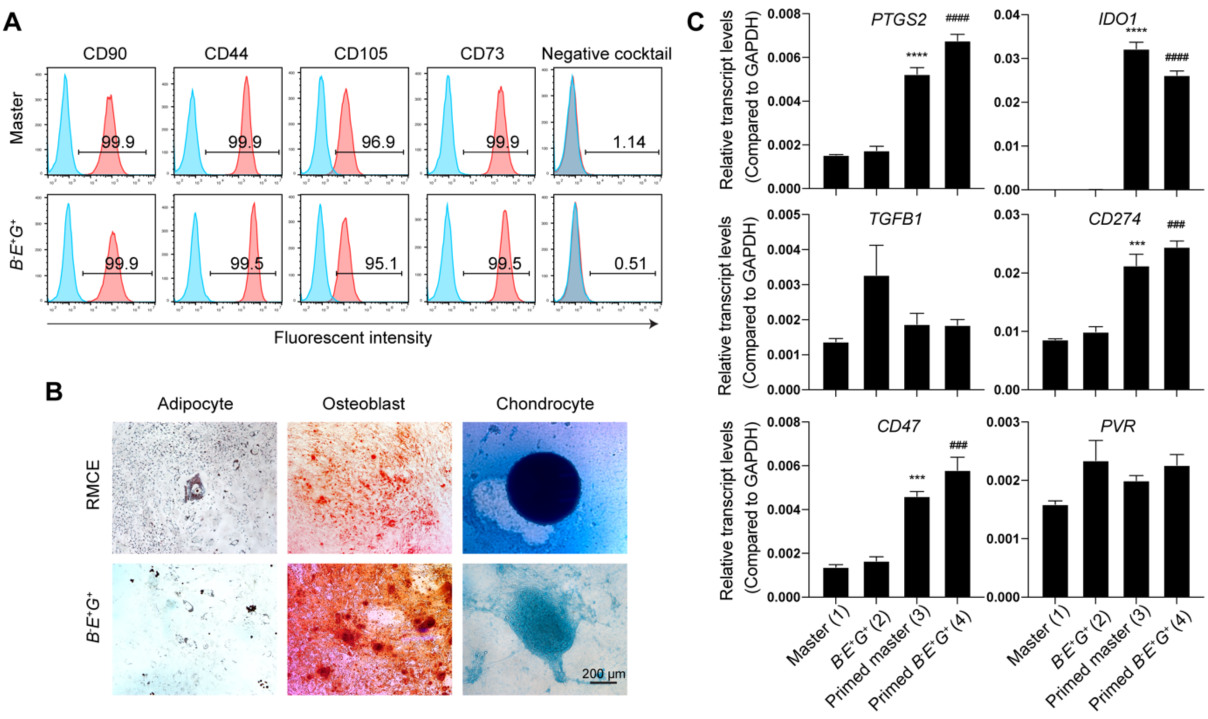

Figure S4. Characterization of master and $B^{-}E^{+}G^{+}$ EMSCs.

(A) Flow cytometry analysis of master and $B^{-}E^{+}G^{+}$ EMSCs for MSC markers and a negative marker cocktail.

(B) Trilineage differentiation from master and $B^{-}E^{+}G^{+}$ EMSCs. Scale bar, 200 μm.

(C) qPCR analysis for expression of immunosuppressive genes in master and $B^{-}E^{+}G^{+}$ EMSCs (naïve and IFN-γ-primed). N = 3. $^{***}P < 0.001$ and $^{****}P < 0.0001$ for (3) versus (1). $^{###}P < 0.001$ and $^{####}P < 0.0001$ for (4) versus (2) per ordinary one-way ANOVA followed by Dunnette's multiple comparison test.

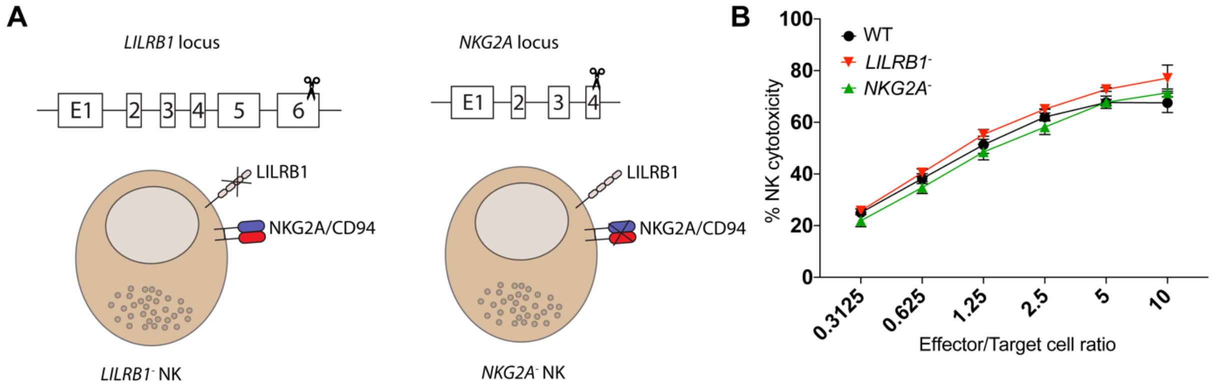

Figure S5. *LILRB1* and *NKG2A* knockout in NK-92MI cells doesn't compromise their cytotoxicity against K562.

(A) Schematic graph for knockout of *LILRB1* (left) and *NKG2A* (right) in NK-92MI cells.

(B) WT, *LILRB1*$^{-}$, and *NKG2A*$^{-}$ NK-92MI cell-mediated lysis of K562. N = 3.

A

Mismatch	Target site	DNA sequence	Chromosome	in genes
	On-target	ACTCTCTCTTTCTGGCCTGGAGG	chr15	*B2M*
3	Off-target 1	cCTCTCTCTTTCgGGCgTGGAGG	chr8	*MROH5*
3	Off-target 2	gCTCTCTCTTTCTGGCtTtGTGG	chr15	*RYR3*
3	Off-target 3	AacCTtTCTTTCTGGCCTGGTGG	chr5	*CWC27*
	On-target	CGCGAGCACAGCTAAGGCCACGG	chr15	*B2M*
2	Off-target 4	CGCGAGCACAGCcgAGGCCATGG	chr10	*GATA3*
3	Off-target 5	CagGAGCACAGCcAAGGCCAGGG	chr5	*FGFR4*
3	Off-target 6	CcCtAGCACAGCTAgGGCCAGGG	chr2	*ALK*

B

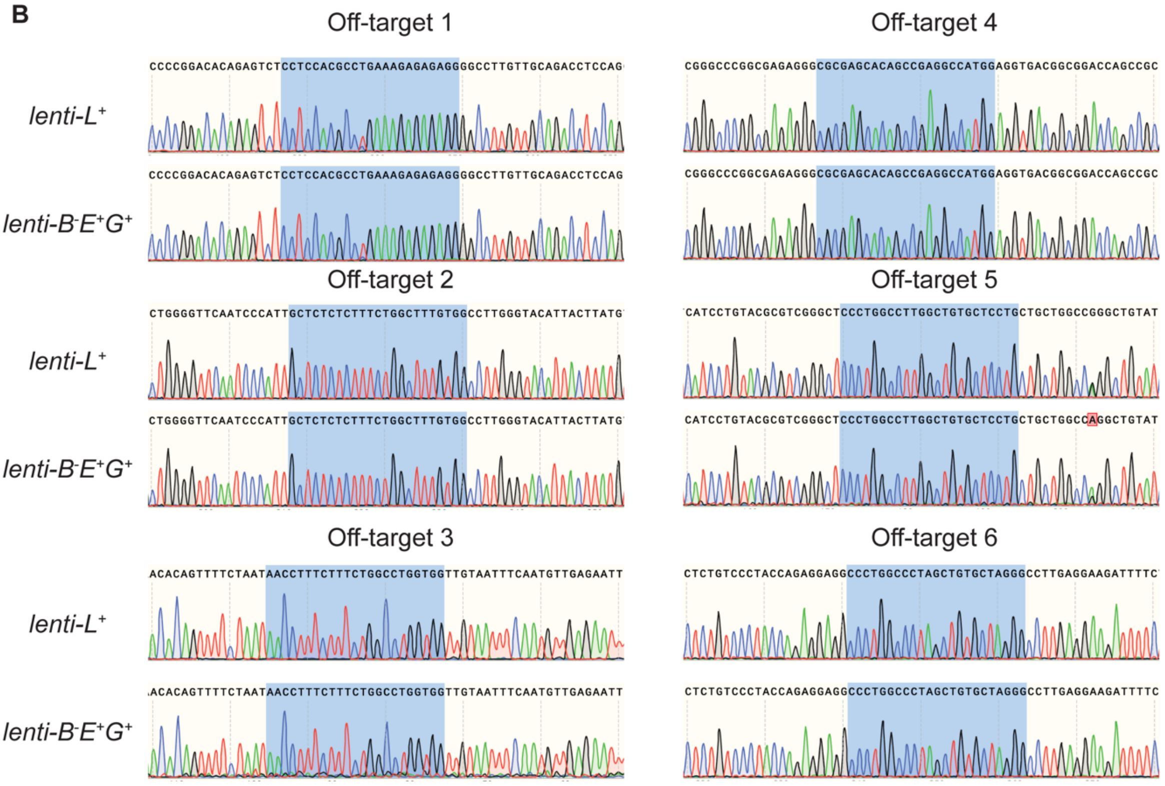

Figure S6. Off-target screening of genetically edited EMSCs.

(A) Information of top 6 potential off-target sites predicted via Cas-OFFinder.

(B) Sanger sequencing results of the predicted off-target sites in *lenti-L*$^{+}$ and *lenti-B*$^{-}$*E*$^{+}$*G*$^{+}$ EMSCs.

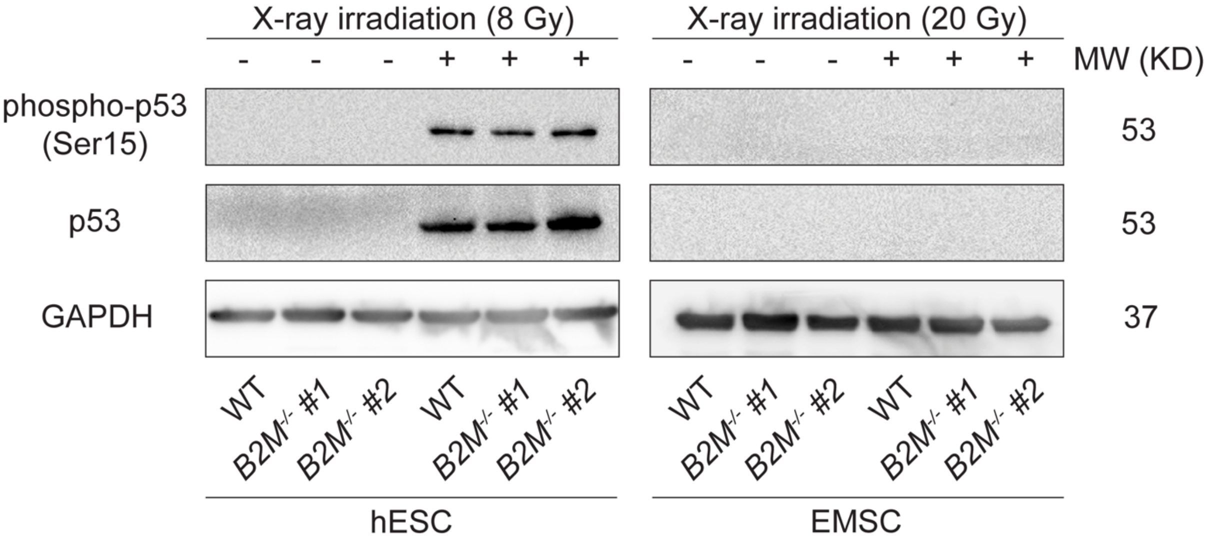

Figure S7. P53 status in genetically manipulated hESCs and EMSCs.

Western blot analysis for total and phosphorylated p53 in WT and *B2M*$^{-/-}$ hESCs (Left) and EMSCs (Right). X-ray irradiation (8 Gy or 20 Gy) used for p53 activation in the cells. GAPDH was used as a loading control.

A

	A	B	C	D	E	F	G	H	I	J	K
1	POS	POS	POS	POS	NEG	NEG	ENA-78 (CXCL5)	G-CSF	GM-CSF	GRO a/b/g	GRO alpha (CXCL1)
2	I-309 (CCL1)	IL-1 alpha (IL-1 F1)	IL-1 beta (IL-1 F2)	IL-2	IL-3	IL-4	IL-5	IL-6	IL-7	IL-8 (CXCL8)	IL-10
3	IL-12 p40/p70	IL-13	IL-15	IFN-gamma	MCP-1 (CCL2)	MCP-2 (CCL8)	MCP-3 (CCL7)	M-CSF	MDC (CCL22)	MIG (CXCL9)	MIP-1 beta (CCL4)
4	MIP-1 delta	RANTES (CCL5)	SCF	SDF-1 alpha	TARC (CCL17)	TGF beta 1	TNF alpha	TNF beta (TNFSF1B)	EGF	IGF-1	Angiogenin
5	OSM	TPO	VEGF-A	PDGF-BB	Leptin	BDNF	BLC (CXCL13)	Ck beta 8-1 (CCL23)	Eotaxin-1 (CCL11)	Eotaxin-2 (CCL24)	Eotaxin-3 (CCL26)
6	FGF-4	FGF-6	FGF-7 (KGF)	FGF-9	FLT-3 Ligand	Fractalkine (CX3CL1)	GCP-2 (CXCL6)	GDNF	HGF	IGFBP-1	IGFBP-2
7	IGFBP-3	IGFBP-4	IL-16	IP-10 (CXCL10)	LIF	LIGHT (TNFSF14)	MCP-4 (CCL13)	MIF	MIP-3 alpha	NAP-2 (CXCL7)	NT-3
8	NT-4	OPN (SPP1)	OPG (TNFRSF11	PARC	PLGF	TGF beta 2	TGF beta 3	TIMP-1	TIMP-2	POS	POS

B

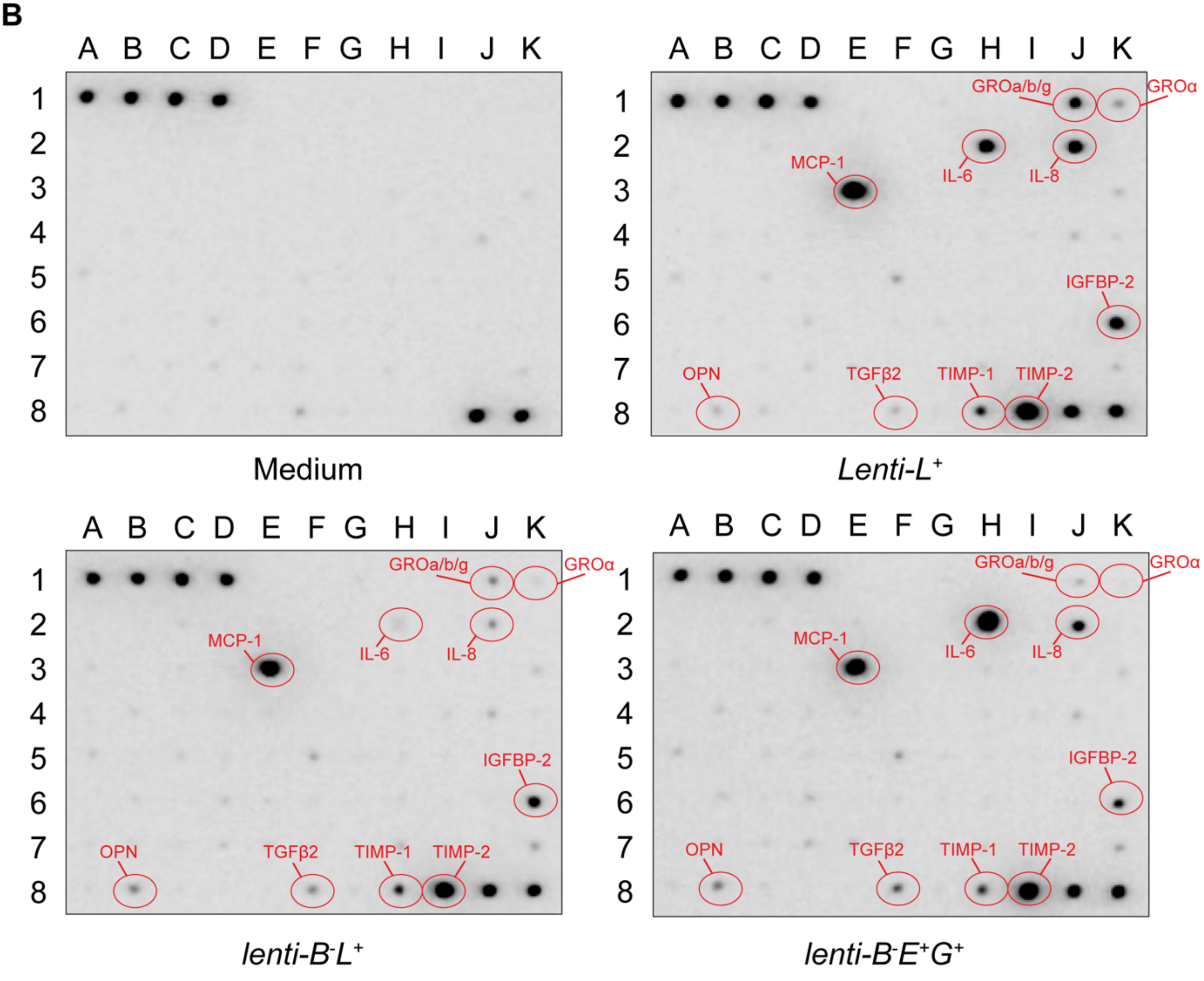

Figure S8. Cytokine expression profiling on genetically manipulated EMSCs.

(A) Layout of the human cytokine antibody array.

(B) Array readouts of fresh αMEM medium and conditioned medium from the culture of *lenti-L*$^{+}$, *lenti-B*$^{-}$*L*$^{+}$, and *lenti-B*$^{-}$*E*$^{+}$*G*$^{+}$ EMSCs.

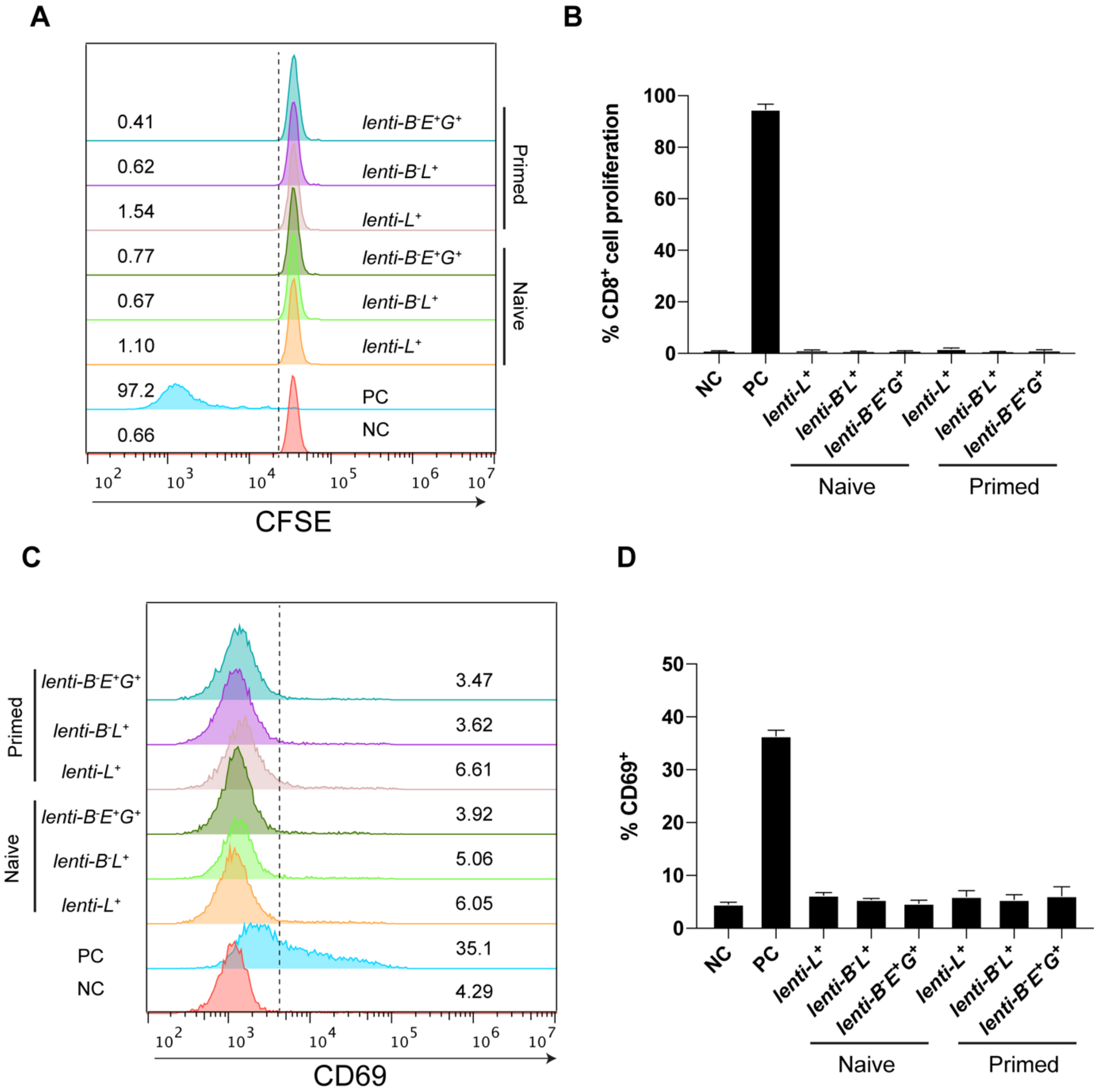

Figure S9. Assay of cytotoxic T cell activation following co-culture with genetically manipulated EMSCs.

(A) Flow cytometry assay of proliferation of CFSE-labelled CD8$^+$ T cells following co-culture with naïve or IFN-γ-primed *lenti-L*$^+$, *lenti-B*$^-$*L*$^+$ and *lenti-B*$^-$*E*$^+$*G*$^+$ EMSCs. PBMCs alone were used as a negative control (NC) and PHA-treated PBMCs a positive control (PC).

(B) Bar graph for the percentage of proliferating CD8$^+$ T cells assayed above. N = 3.

(C) Flow cytometry assay for $CD69^+$ cells among $CD8^+$ T cells co-cultured with EMSCs as above.

(D) Bar graph for the percentage of $CD69^+CD8^+$ T cells assayed above. N = 3.

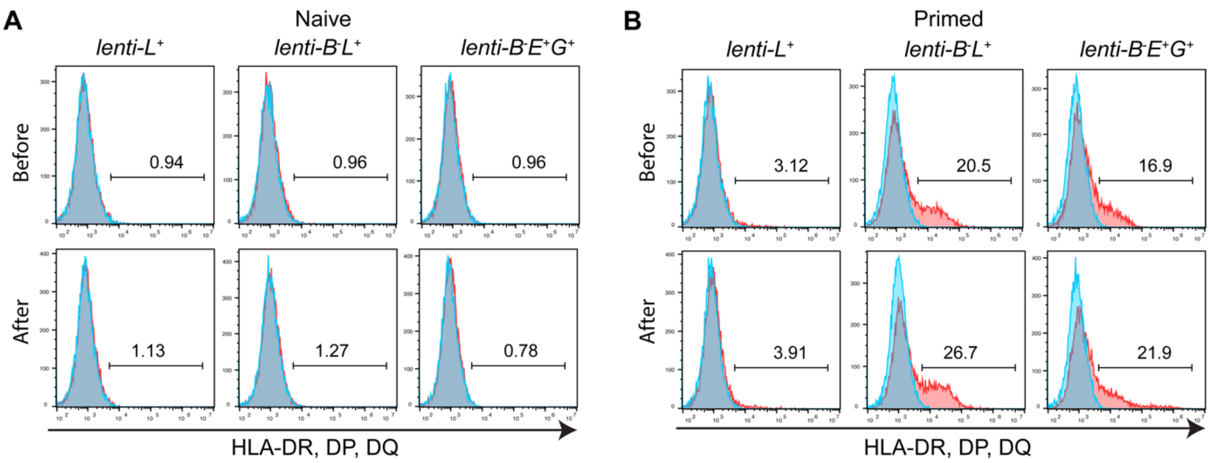

Figure S10. HLA class II expression on genetically manipulated EMSCs before and after co-culture with NK cells.

Surface expression of HLA class II on naïve (A) or IFN-γ-primed (B) *lenti-L*$^{+}$, *lenti-B*$^{-}$*L*$^{+}$, and *lenti-B*$^{-}$*E*$^{+}$*G*$^{+}$ EMSCs before and after co-culture with NK-92MI cells.
